# 3D‐Printable Nanoporous Thermosets via Disulfide‐Based Polymerization‐Induced Microphase Separation

**DOI:** 10.1002/anie.8227484

**Published:** 2026-06-13

**Authors:** Xueheng Dai, Kenny Lee, Yuan Xiu, Nathaniel Corrigan, Kun Zhou, Xichuan Li, Christopher M. Bates, Craig J. Hawker, Cyrille Boyer

**Affiliations:** ^1^ Cluster for Advanced Macromolecular Design (CAMD) and Australian Centre for Nanomedicine (ACN) School of Chemical Engineering University of New South Wales Sydney NSW Australia; ^2^ Materials Research Laboratory and Materials Department University of California Santa Barbara Santa Barbara California USA; ^3^ Centre for Advanced Manufacturing Technology (CfAMT) School of Engineering Western Sydney University Sydney NSW Australia

**Keywords:** dynamic disulfide, LCD 3D printing, nanoporous material, polymer degradation, α‐lipoic acid

## Abstract

Interconnected nanoporous polymer networks are central to applications that demand rapid mass transport, yet their fabrication by polymerization‐induced microphase separation (PIMS) remains constrained by fixed macroCTA polarity, limited formulation compatibility, and difficult translation to additive manufacturing. Here, we address these limitations by introducing a PIMS strategy utilizing chemically degradable macroCTAs with systematically tunable hydrophilicity. These macroCTAs were synthesized via reversible addition−fragmentation chain‐transfer copolymerization of α‐lipoic acid or ethyl lipoate with various acrylates. This diverse library of macroCTAs enabled the preparation of microphase‐separated materials across a broad range of monomer and crosslinker chemistries, which were readily converted into nanoporous thermosets with well‐defined pore sizes (24–42 nm) via selective disulfide cleavage. Critically, these photocurable resins are compatible with liquid‐crystal display 3D printing, allowing the fabrication of complex, hierarchical architectures that can be directly etched to generate embedded nanoscale porosity while preserving structural integrity. Collectively, tunable and degradable macroCTAs bridge 3D‐printable form factors with programmable nanoscale structure, providing a general route to hierarchically structured materials for separations, catalysis, and advanced manufacturing.

## Graphical abstract

α‐Lipoic acid‐derived degradable macro‐chain transfer agents (macroCTAs) with tunable hydrophilicity drive polymerization‐induced microphase separation (PIMS) in photocurable resins. Selective etching via disulfide cleavage then generates interconnected nanoporous thermosets, and compatibility with liquid‐crystal display (LCD) 3D printing enables the fabrication of complex architectures with tunable mesoporosity.

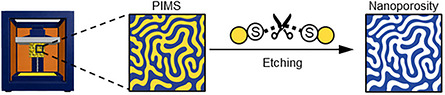

## 1 Introduction

Nanoporous polymer materials underpin a wide range of mass‐transport technologies, from energy storage and conversion to separations and catalysis [1, 2, 3, 4, 5, 6, 7, 8, 9, 10], by providing a high internal surface area and interconnected pathways for molecular and ionic transport. This utility is further amplified by rapid advancements in nanotechnology and an increasing global commitment to improving resource and energy sustainability [11]. Among various synthetic strategies, polymerization‐induced microphase separation (PIMS) has emerged as an efficient and tunable approach for preparing robust nanoporous networks [12, 13, 14, 15, 16, 17, 18, 19, 20]. In a typical PIMS system, polymerization results in the in situ formation of a block copolymer consisting of two chemically distinct domains: a non‐crosslinked macro‐chain transfer agent (macroCTA) domain and a rigid, crosslinked matrix [21]. The incompatibility of the macroCTA with the polymerizing monomers/crosslinkers constituting the second block drives a competition between microphase separation and crosslinking that results in controllable, nanometer‐sized channels [22, 23, 24, 25]. By utilizing a macroCTA designed for selective etching, this continuous domain can be removed without compromising the mechanical integrity of the surrounding matrix, effectively transforming a PIMS monolith into a material with a highly interconnected pore network [12, 13, 14, 21].

**FIGURE 1:**
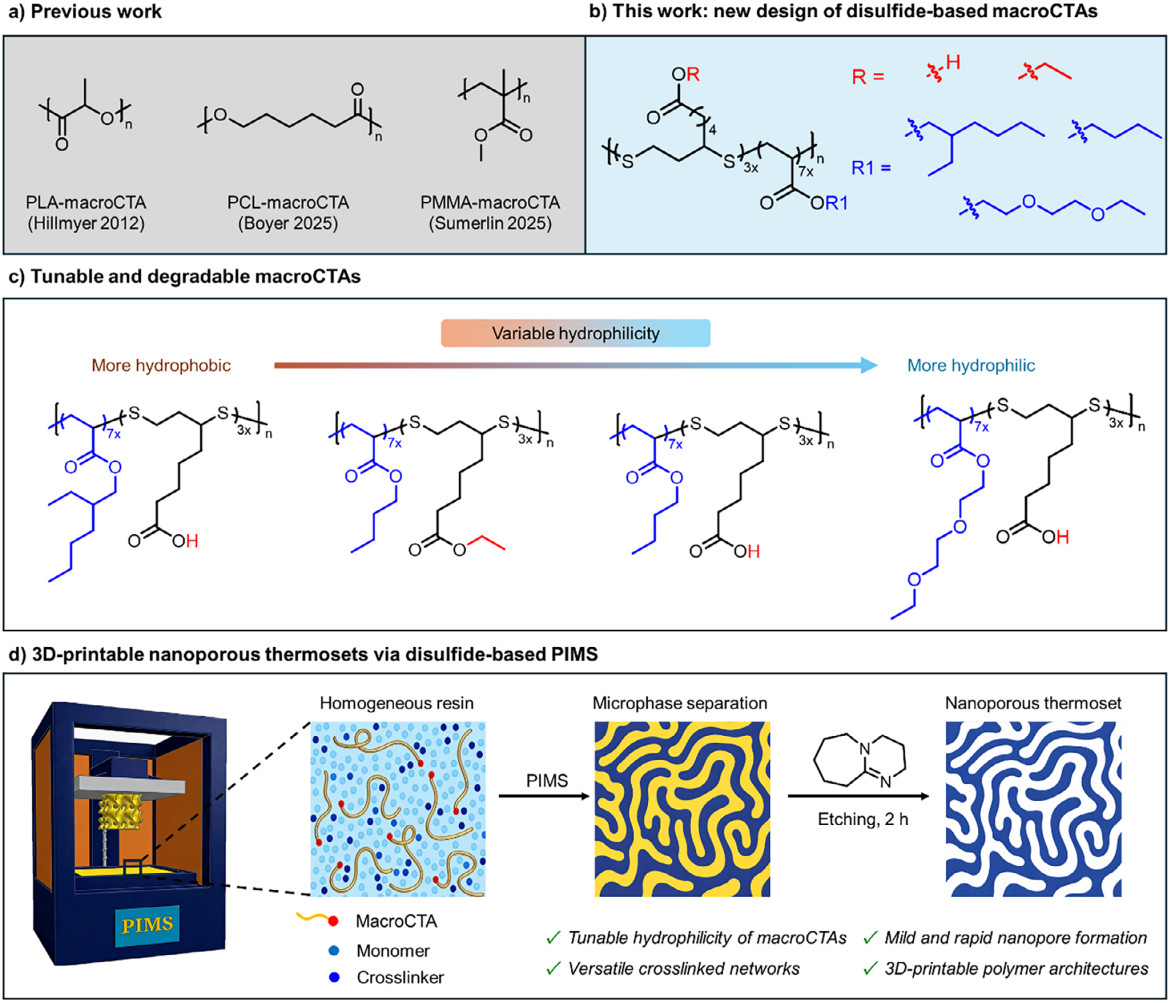
Comparative overview of PIMS strategies used for fabricating nanoporous materials. (a) Previous work: chemical structures of PLA, PCL, and PMMA macroCTAs, previously used in PIMS for the fabrication of nanoporous materials [12, 15, 16]. (b–d) This work: (b and c) chemical structures of degradable disulfide‐based macroCTAs with variable hydrophilicity, and (d) schematic representation of the PIMS process highlighting the ability to fabricate 3D‐printed nanoporous materials with tunable porosity.

A diverse library of macroCTAs has been explored to tune these pore characteristics and the overall composition of the crosslinked porous matrix, as exemplified by the representative macroCTA designs shown in Figure 1a. For example, Seo and Hillmyer pioneered the PIMS concept by carrying out thermally initiated reversible addition–fragmentation chain‐transfer (RAFT) copolymerization of styrene (S) and divinylbenzene (DVB) in the presence of a poly(lactic acid) chain‐transfer agent (PLA‐CTA), generating PLA‐*b*‐P(S‐*co*‐DVB) block copolymers that underwent microphase separation to form well‐defined morphologies [12, 26]. Subsequent alkaline etching of PLA yielded mesoporous materials with pore diameters ranging from 4 nm to 8 nm. This concept was further adapted to a photoinitiated PIMS system by Seo and coworkers, who utilized UV photopolymerization to drive the copolymerization of isobornyl acrylate (IBoA) and ethylene glycol diacrylate in the presence of PLA‐CTA [17]. In a similar vein, our group used polycaprolactone (PCL) as a macroCTA to mediate the photopolymerization of IBoA with poly(ethylene glycol) diacrylate (*M*_n_ = 575 g mol^−1^). In this system, nanoporous materials were achieved through selective PCL degradation in aqueous NaOH at 60°C for 2 days [15]. More recently, shifting away from chemical etching, Sumerlin and coworkers developed nanoporous thermosets via thermal depolymerization of poly(methyl methacrylate) (PMMA). By heating PMMA within a poly(S‐*co*‐DVB) matrix to 290 °C, they generated mesoporous structures with high surface areas of 200 m^2^ g^−1^ [16].

Despite these advantages, conventional PIMS approaches are constrained by three interrelated limitations. First, the selection of the macroCTA is typically restricted to hydrophobic polymers (such as PLA, PCL, or PMMA), and their hydrophilicity cannot be easily tuned without reformulating the entire system. Second, the crosslinked matrix is generally confined to a narrow range of styrenic or acrylic monomers, restricting the achievable glass transition temperature (*T*_g_), mechanical properties, and curing modality of the domain supporting the porous network. Third, existing etching protocols, such as high‐temperature depolymerization (290 °C) or prolonged alkaline treatment (60 °C for 2 days), are often incompatible with photocured monoliths [27, 28]. Collectively, these constraints restrict the design of nanoporous materials, particularly for applications requiring both tailored porosity and complex geometries.

Recent advances in 3D printing have increasingly leveraged dynamic or degradable polymer networks to impart self‐healing, reprocessability, and controlled degradability to printed materials. Dynamic covalent bonds, including disulfides, imines, and boronate esters, have been incorporated into photocurable resins and hydrogels, enabling post‐printing repair, stress relaxation, and on‐demand degradation [29, 30, 31, 32]. Lipoate‐based photopolymer systems have further demonstrated the feasibility of fully recyclable 3D‐printed objects through reversible thiol–disulfide exchange under mild conditions [30, 33]. Despite these advances, existing approaches predominantly target macroscopic material properties， such as mechanical recovery and bulk reusability, and do not exploit the selective removal of degradable domains to generate nanoscale porosity within the printed architectures. The use of sacrificial, phase‐separated domains as nanopore templates in 3D‐printed objects, as realized here through the integration of PIMS with light‐based additive manufacturing, therefore remains largely unexplored and represents a conceptually distinct strategy for introducing functional nanoscale architectures into complex printed geometries.

To address these limitations, we identified α‐lipoic acid (LA) as an attractive building block for the preparation of porous materials. The 1,2‐dithiolane ring present in LA provides a straightforward handle for introducing chemically cleavable disulfide bonds into the etchable domain by synthesizing macroCTAs via radical ring‐opening copolymerization with vinyl monomers [34, 35, 36, 37, 38, 39, 40, 41, 42, 43, 44]. Unlike traditional hydrophobic macroCTAs, LA‑based systems are readily compatible with RAFT copolymerization, allowing for precise control over copolymer composition, hydrophilicity, and molecular weight [45, 46]. Recent studies have shown that LA efficiently copolymerizes with acrylates or acrylamides to afford well‐defined LA–vinyl copolymers with narrow molar‐mass distributions, which can subsequently be degraded via selective disulfide cleavage of LA–LA diads along the polymer backbone [47]. Building on this concept, we synthesized a family of degradable macroCTAs derived from LA or its ethyl ester analogue, ethyl lipoate (ELp), in combination with acrylates of systematically varied hydrophilicity (Figure 1b). These macroCTAs were formulated with a range of vinyl monomers and crosslinkers to produce photocurable resins that could be cured either in bulk or using liquid‐crystal display (LCD) 3D printing under 405 nm irradiation. Subsequent base‐catalyzed disulfide cleavage selectively removed the macroCTA domains, converting the PIMS precursors into nanoporous thermosets with interconnected pore networks. This disulfide‐enabled PIMS strategy is particularly powerful for additive manufacturing: complex 3D architectures can be printed first and then chemically etched to introduce tunable nanoscale porosity, directly linking macroscopic design freedom with high‐surface‐area materials for mass‐transport applications.

## 2 Results and Discussion

### 2.1 Synthesis and Characterization of Disulfide‐Based MacroCTAs

**FIGURE 2:**
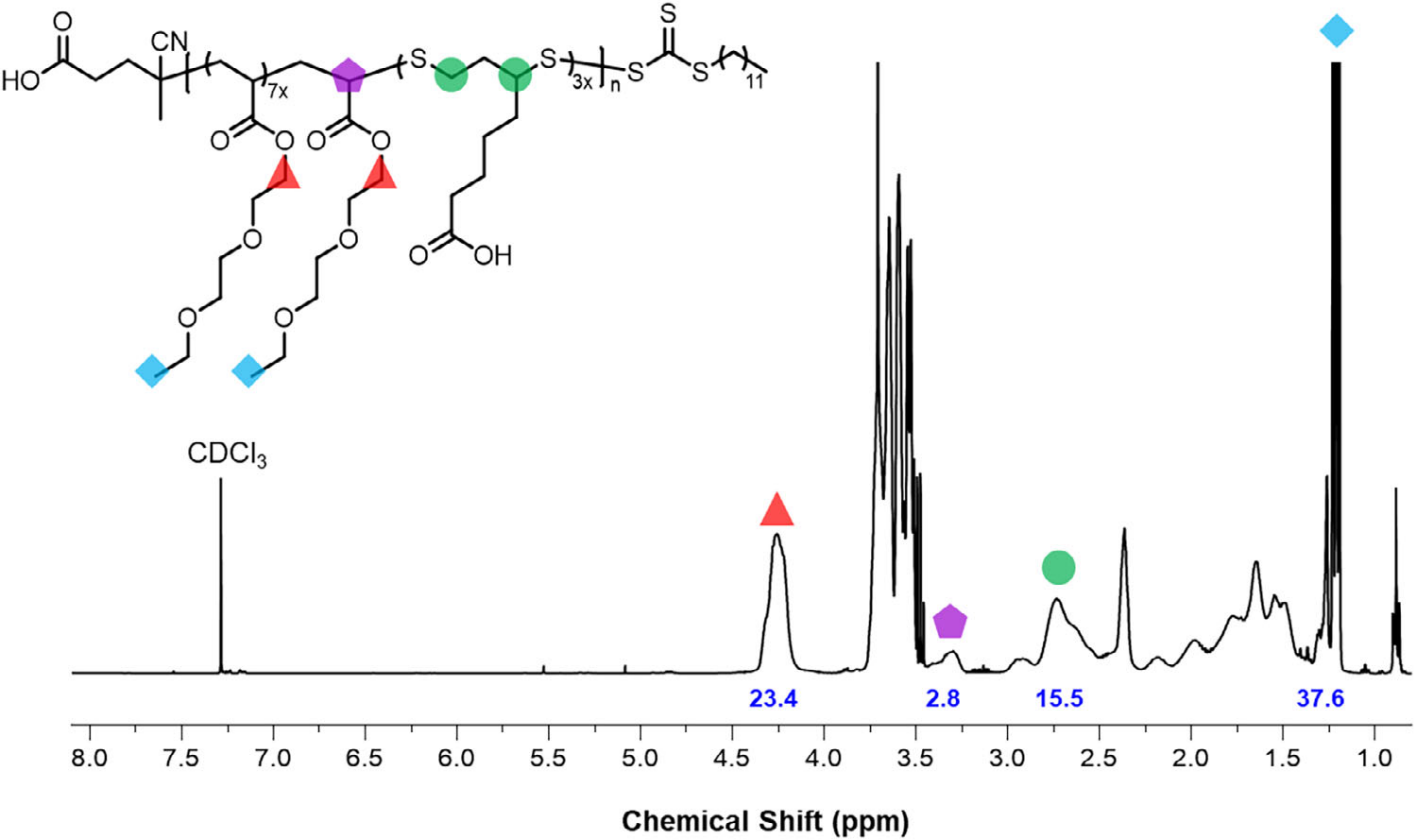
^1^H NMR spectrum of purified P(LA‐*co*‐EEA)‐macroCTA in CDCl_3_ (400 MHz). Quantification of LA incorporation was achieved by integration of thioether signals (2.50–2.80 ppm, green circle) from the ring‐opened LA units relative to the CH_2_ ester signal of EEA side‐chain methylene protons (4.10–4.40 ppm, red triangle). The thioether signal intensity and broadening confirmed dithiolane ring‐opening during copolymerization. Methine resonances at 3.25–3.45 ppm (purple pentagon) from backbone carbons adjacent to LA units are consistent with true copolymer formation rather than a mixture of homopolymers. The chemical structure shows the ring‐opened LA repeat unit with disulfide linkages incorporated into the polymer backbone.

To implement our degradable‐macroCTA PIMS strategy, a family of LA‐ and ELp‐derived macroCTAs was prepared by thermal RAFT polymerization (Scheme S1). LA and its ethyl ester derivative ELp were incorporated as complementary lipoate‐based monomers to enable systematic tuning of macroCTA polarity across a broad range. The carboxylic acid group in LA confers relatively high polarity and hydrophilicity, whereas esterification in ELp renders the monomer considerably more hydrophobic. Rather than representing alternative choices, LA and ELp were each paired with acrylate comonomers of varying polarity to access distinct regions of the hydrophilicity window that would not be achievable with a single lipoate monomer alone. Since macroCTA polarity is a key parameter governing the thermodynamic driving force for microphase separation and the resulting domain spacing in PIMS systems, this combinatorial approach maximizes the range of accessible nanoporous morphologies and enables systematic control over pore dimensions in the resulting nanoporous thermosets. As a precursor to the ELp series, ELp was first synthesized via 1‐(3‐dimethylaminopropyl)‐3‐ethylcarbodiimide hydrochloride (EDC·HCl) coupling of LA with ethanol (Figure S1). LA or ELp was copolymerized with vinyl acrylate comonomers using 4‐cyano‐4‐(((dodecylthio)carbonothioyl)thio)pentanoic acid (CDTPA) as the chain‐transfer agent (CTA) and 2,2′‐azobisisobutyronitrile (AIBN) as the thermal initiator. The LA or ELp feed fraction was fixed at 30 mol%, a choice made to balance a narrow molecular weight distribution with high disulfide content, thereby ensuring optimal degradability [34]. Three acrylate comonomers spanning a systematic range of polarity—2‐ethylhexyl acrylate (EHA), *n*‐butyl acrylate (*n*‐BA), and 2‐(2‐ethoxyethoxy)ethyl acrylate (EEA)—were selected to tune macroCTA hydrophilicity. These monomers enabled the preparation of P(LA‐*co*‐EHA), P(ELp‐*co*‐BA), P(LA‐*co*‐BA), and P(LA‐*co*‐EEA) macroCTAs (Figure 1c), providing a systematic means of tuning macroCTA hydrophilicity and, consequently, the microphase‐separated morphology and pore dimensions of the resulting nanoporous materials. Polymerizations were stopped at 70%–80% monomer conversion to maintain good control over molar mass and dispersity (*Đ*) (Table 1 and Figure S2) [48]. After polymerization, the copolymers were purified by precipitation to eliminate residual monomers and analyzed by ^1^H NMR spectroscopy to confirm the successful incorporation of LA or ELp (Figure 2). The formation of copolymers, rather than simple homopolymer blends, was confirmed by the appearance of characteristic methine signals at 3.25–3.45 ppm, which are assigned to acrylate protons (CH) adjacent to LA or ELp units [39]. Quantitative analysis by integration of the thioether resonances at 2.50–2.80 ppm attributed to ELp or LA repeat units revealed contents of 28–34 mol% in the polymer backbone, in excellent agreement with the feed ratio of 30 mol% (Figures S3–S5) [39].

**TABLE 1:** Composition and molecular weight characteristics of the synthesized macroCTAs before and after degradation.

Entry	Disulfide monomer	Acrylate comonomer	Disulfide feed (%)	Acrylate mol%^a^	Disulfide mol%^a^	*M*_n,SEC_ ^b^	*Đ* ^b^	*M*_n,deg_ ^c^
1	LA	EHA	30	66	34	21	1.20	5.1
2	ELp	*n*‐BA	30	71	29	16	1.18	5.4
3	LA	*n*‐BA	30	72	28	24	1.20	6.3
4	LA	EEA	30	69	31	7.7	1.21	2.8

^a^ Determined by ^1^H NMR spectroscopy from integration of characteristic resonances.

^b^ *M*_n_ and *Đ* determined in THF SEC calibrated with PMMA standards (*M*_n,SEC_ in kg mol^−1^).

^c^ *M*_n,deg_ determined by THF SEC after 2 h of DBU‐catalyzed degradation (*M*_n,deg_ in kg mol^−1^).

**FIGURE 3:**
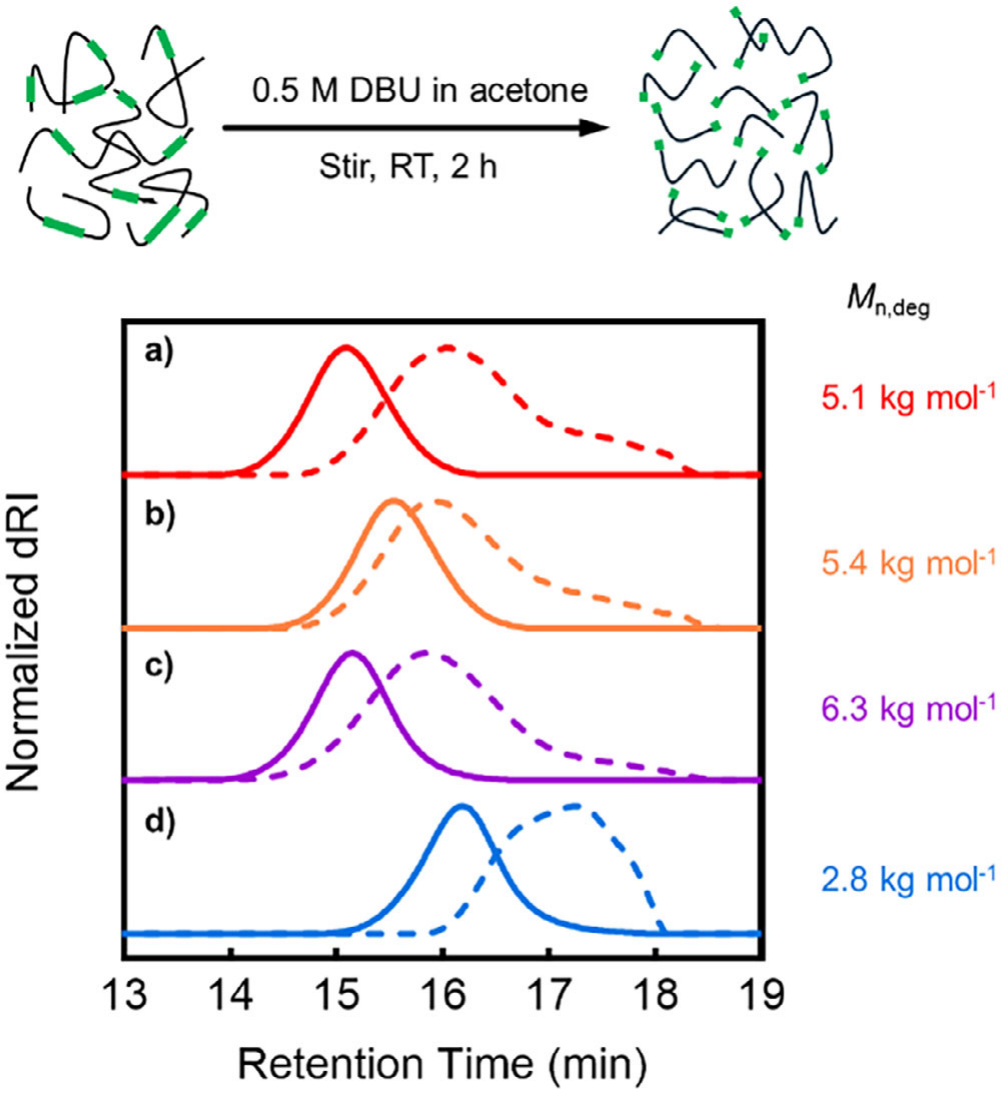
SEC traces demonstrating the DBU‐catalyzed degradation of disulfide‐based macroCTAs. Solid lines: purified macroCTAs before degradation; dashed lines: after treatment with 0.5 M DBU in acetone. (a) P(LA‐*co*‐EHA)‐macroCTA, (21→5.1 kg mol^−1^), (b) P(ELp‐*co*‐BA)‐macroCTA, (16→5.4 kg mol^−1^), (c) P(LA‐*co*‐BA)‐macroCTA, (24→6.3 kg mol^−1^), and (d) P(LA‐*co*‐EEA)‐macroCTA, (7.7→2.8 kg mol^−1^). Progressive degradation into lower‐molar‐mass oligomers confirms efficient disulfide cleavage under mild basic conditions, validating the macroCTA design for selective PIMS etching.

Before incorporating these disulfide‐containing macroCTAs into PIMS resins, their chemical cleavability was first validated under the intended etching conditions. Accordingly, macroCTAs were treated with 1,8‐diazabicyclo(5.4.0)undec‐7‐ene (DBU) in acetone at room temperature to promote base‐catalyzed thiol‐disulfide exchange [33, 40]. These reductive conditions target the linear disulfide bonds distributed along the polymer backbone, effectively cleaving the macroCTA into smaller fragments. All macroCTAs were treated with 0.5 M DBU in acetone, and aliquots were withdrawn, quenched with water to deactivate DBU, and diluted in THF for size‐exclusion chromatography (SEC) analysis (Figure S6). A shift of the elution peak toward higher retention volumes, corresponding to a substantial decrease in molecular weight, confirmed their successful degradation (Figure 3). These results validate the macroCTA design and establish the feasibility of DBU‐catalyzed etching for the PIMS system. After a reaction period of 2 h, the degraded samples were analyzed by SEC to determine the evolution of their *M*_n_ and *Đ* (Table 1).

### 2.2 Resin Preparation and PIMS Precursor Formation

In this PIMS framework, the resulting microphase‐separated morphology and domain spacing are primarily set by two parameters: the Flory–Huggins interaction parameter (*χ*), which captures the thermodynamic incompatibility between the degradable macroCTA block and the forming crosslinked matrix, and the effective degree of polymerization (*N*) of the block copolymer generated during curing, which is related to the macroCTA chain length and its loading in the formulation [20, 21, 49, 50]. To target a bicontinuous microphase‐separated morphology, we fixed the macroCTA loading at 30 wt% across all formulations [22]. To ensure a robust, crosslinked matrix, we utilized a 4:1 molar ratio of vinyl monomer to multifunctional acrylate crosslinker. The photoinitiator diphenyl(2,4,6‐trimethylbenzoyl)phosphine oxide (TPO) was fixed at 0.5 wt% to yield rapid photocuring in air.

The crosslinked networks were designed based on three criteria: (i) high *T*_g_ to ensure a rigid, glassy matrix that maintains structural integrity during printing and etching; (ii) good miscibility of monomer and crosslinker with macroCTAs in the resin to ensure homogeneity; and (iii) sufficient chemical dissimilarity between the macroCTA and the forming crosslinked phase to achieve high *χ* and promote efficient microphase separation during photopolymerization [51]. Based on these criteria, four photocurable resins were prepared: (i) P(LA‐*co*‐EHA)‐macroCTA with *N*,*N*‐diethylacrylamide (DEAm) and poly(ethylene glycol) diacrylate (PEGDA, *M*_n_ = 250 g mol^−1^); (ii) P(ELp‐*co*‐BA)‐macroCTA with acrylic acid (AA) and PEGDA; (iii) P(LA‐*co*‐BA)‐macroCTA with AA and PEGDA; (iv) P(LA‐*co*‐EEA)‐macroCTA with IBoA and 1,6‐hexanediol diacrylate (HDDA). The selection of the crosslinker (PEGDA vs. HDDA) was guided by miscibility considerations with the respective macroCTA and monomer combinations. Detailed compositions of each photocurable resin are provided in Table S1.

Subsequently, all formulations yielded transparent, homogeneous resins, confirming good miscibility prior to photocuring. The PIMS precursor resins were stored in sealed containers under dark conditions at 4 °C and exhibited no observable changes in viscosity or printability over a period of at least 2 weeks, demonstrating good storage stability in the absence of external stimuli for disulfide bond exchange. Upon 405 nm irradiation, the resins underwent rapid photopolymerization to afford light‐yellow, optically transparent monoliths. During photopolymerization, chain extension from the macroCTA trithiocarbonate end groups generates growing polymer chains that become progressively more incompatible with the macroCTA segments, driving microphase separation. Simultaneously, crosslinking via the diacrylate monomer kinetically arrests the evolving microphase‐separated morphology, yielding a bicontinuous macroCTA domain and a crosslinked matrix [20]. For clarity, the cured PIMS precursors are denoted PIMS‐EHA, PIMS‐ELp, PIMS‐BA, and PIMS‐EEA, respectively. After etching, the corresponding nanoporous thermosets are referred to as NPT‐EHA, NPT‐ELp, NPT‐BA, and NPT‐EEA, respectively.

### 2.3 Microphase Separation Characterization

**FIGURE 4:**
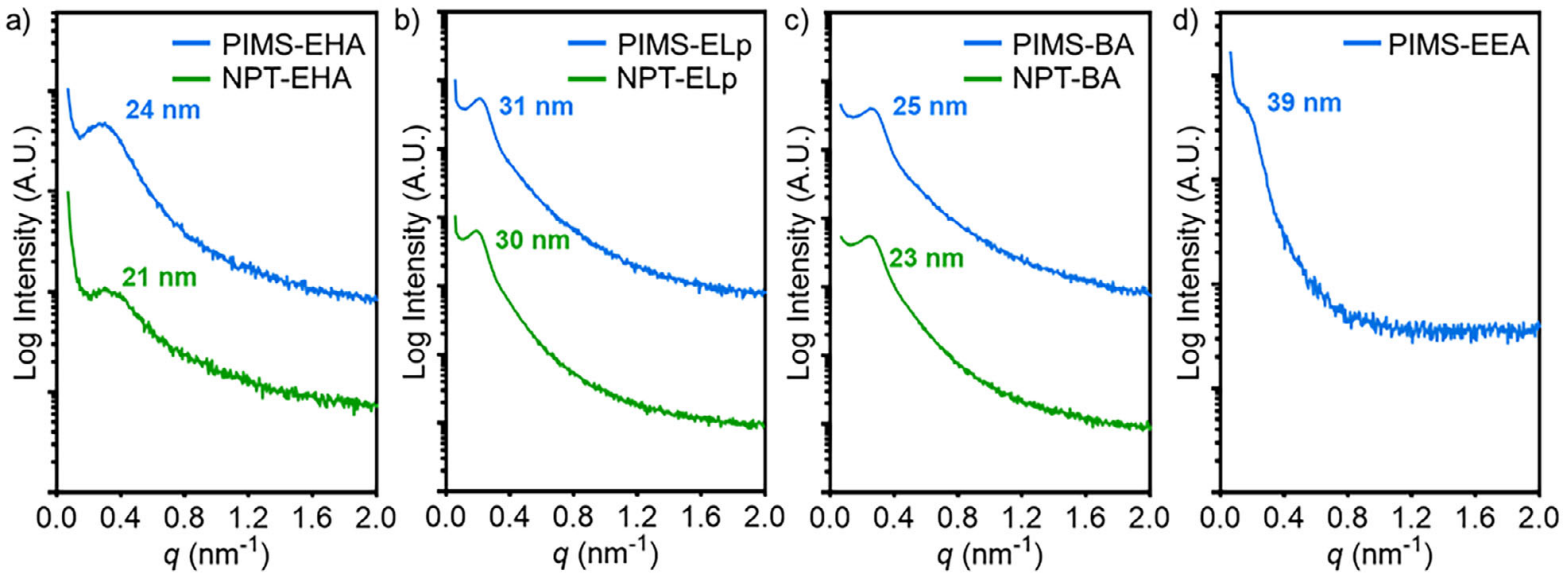
SAXS profiles of PIMS precursors (blue) and nanoporous materials after etching (green). (a) PIMS‐EHA/NPT‐EHA; (b) PIMS‐ELp/NPT‐ELp; (c) PIMS‐BA/NPT‐BA; and (d) PIMS‐EEA. The primary peaks are preserved after etching, confirming the retention of domain spacing without pore collapse.

With these *χ* and *N* design variables established through macroCTA structure and loading, the internal nanostructure and interdomain spacing of the resulting PIMS precursors were quantified by small‐angle X‐ray scattering (SAXS). Each resin was processed using a commercial LCD 3D printer (*λ*_max_ *=* 405 nm, *I*_0_ *=* 3.5 mW cm^−2^) to fabricate square prisms (6 × 6 × 0.2 mm). Figure 4 shows SAXS profiles of the printed PIMS monoliths. All samples exhibit a single broad scattering peak characteristic of a disordered, microphase‐separated morphology [12, 13]. This lack of long‐range order is attributed to the kinetic arrest by rapid in situ crosslinking during the 3D printing process, a phenomenon consistent with previous PIMS reports [22, 52]. Depending on the macroCTA, the primary scattering peak position varied from q^*^ = 0.16–0.30 nm^−1^, corresponding to domain spacings (*d*_SAXS_ = 2π/q^*^) ranging from 24 nm to 39 nm. For example, PIMS using P(LA‐*co*‐EEA)‐macroCTA exhibited the largest interdomain spacing (*d* = 39 nm), while the smallest interdomain spacing was obtained using P(LA‐*co*‐EHA)‐macroCTA (*d* = 24 nm) despite the relatively high *N* (*M*_n_ = 21 kg mol^−1^).

Dynamic mechanical analysis (DMA) was performed on the 3D‐printed PIMS precursors (30 × 8 × 2 mm) to evaluate thermomechanical stability. The tan *δ* profiles (Figure S7) exhibited two distinct peaks at approximately −50°C to −20°C and 50°C to 100°C associated with the glass transitions of the disulfide‐containing macroCTA domain and the crosslinked matrix, respectively, thus confirming the microphase‐separated nanostructure observed by SAXS. A drop in the storage modulus (*G′*) was also observed near −30 °C due to softening of the macroCTA domain (Figure S8), followed by a gradual decrease in *G′* continued with increasing temperature until the materials softened around 50 °C–80 °C, likely due to passing through the glass transition of the crosslinked network. The glass transition temperatures of these networks, extracted from the tan *δ* peak maxima, ranged from 53°C to 81°C across the different formulations, confirming sufficient thermal stability for the room‐temperature etching and solvent drying steps employed in this work.

### 2.4 Selective Etching and Nanopore Formation

Building upon the successful degradation of the macroCTAs and the confirmation of microphase separation, we investigated the selective etching of 3D‐printed monoliths to generate nanoporous materials. Solvent selection proved critical for achieving well‐defined nanoporous structures, as the etching solvent must sufficiently swell the crosslinked network to facilitate DBU diffusion into the macroCTA domains while ensuring the matrix retains enough mechanical integrity to prevent irreversible deformation [53]. Selective etching was performed by immersing the PIMS monoliths in 0.5 M DBU/solvent solutions for 2 h at room temperature. To optimize this process, we evaluated solvents across a range of polarities, including toluene, acetone, and methanol. In the case of toluene (nonpolar, dielectric constant *ε* = 2.4) containing 0.5 M DBU, no appreciable mass loss was observed by gravimetry after 2 h, likely due to limited network swelling and the poor solubility of DBU in nonpolar media. Conversely, highly polar solvents, such as methanol (*ε* = 32.7), caused excessive swelling, leading to monolith fragmentation and structural cracking (Figure S9). Acetone, a moderately polar solvent (*ε* = 20.7), provided the optimal balance, offering moderate network swelling, enabling DBU to penetrate the monolith while promoting efficient diffusion of soluble degradation products out of the matrix [54]. The low boiling point of acetone also enabled rapid removal by vacuum drying at 40 °C without perturbing the developing pore structure. Accordingly, acetone was selected as the standard etching solvent for all subsequent experiments. Following the etching process in acetone, the monoliths were washed repeatedly with fresh acetone and vacuum dried to a constant mass. Gravimetric analysis showed mass losses of 26–29 wt% across all specimens, in close agreement with the nominal 30 wt% macroCTA loading and consistent with nearly complete removal of the degradable macroCTA domains (Table S2). These consistent results across different formulations, regardless of variations in macroCTA hydrophilicity, confirmed that the disulfide‐rich domains are uniformly accessible throughout the bulk of the material. More importantly, the etched monoliths largely retained their macroscopic dimensions and overall structural integrity, with no obvious cracking or macroscopic deformation following etching. An etching time of 2 h was found to provide an optimal balance between efficient macroCTA removal and structural preservation: shorter durations resulted in incomplete mass loss, whereas prolonged exposure led to excessive swelling and occasional structural fragmentation.

### 2.5 Structural Evolution from PIMS Materials to Nanoporous Networks

To track the structural evolution from PIMS materials to nanoporous monoliths, SAXS and scanning electron microscopy (SEM) were performed following selective etching. The SAXS profiles of etched monoliths closely matched those of the corresponding precursors; the primary scattering peaks appeared at nearly identical values of the primary scattering peak q*, with only minor variations in intensity and peak width (Figure 4). Quantitative analysis showed that the peak positions shifted by less than 15% upon etching, indicating that the interdomain spacing decreased only slightly. For instance, PIMS‐EHA displayed an interdomain spacing of approximately 24 nm before etching, which reduced to roughly 21 nm afterward. This minor contraction suggests that the network remained structurally stable throughout the etching process, avoiding significant pore collapse or reorganization [52, 53].

**FIGURE 5:**
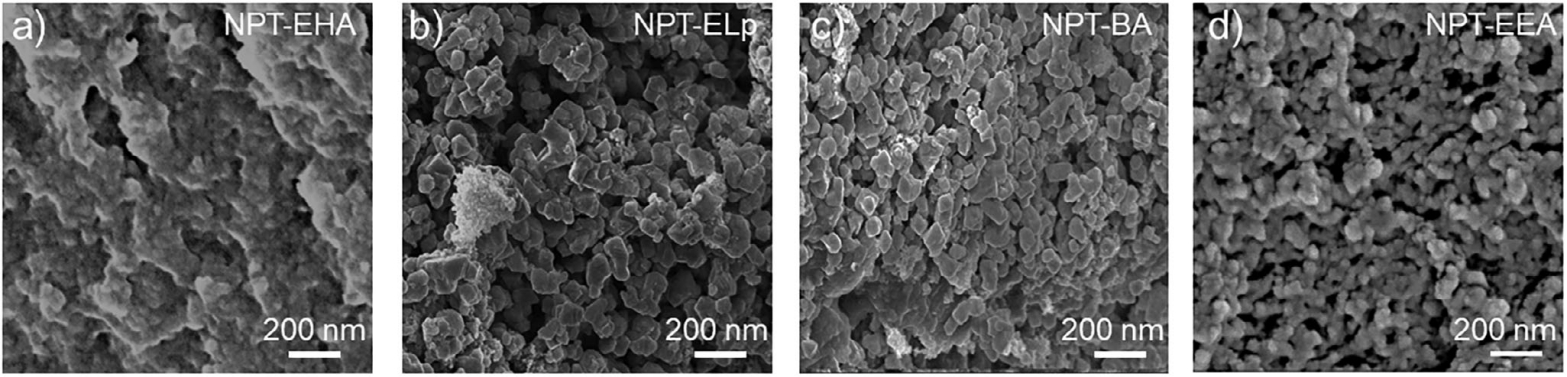
Cross‐sectional SEM images of nanoporous thermosets obtained after selective etching of the disulfide‐containing macroCTAs. (a) NPT‐EHA; (b) NPT‐ELp; (c) NPT‐BA; and (d) NPT‐EEA. Scale bars: 200 nm. The Supporting Information shows the cross‐sectional SEM images before etching.

To visualize the nanoscale morphology, cross‐sectioned fracture surfaces were examined by SEM with the PIMS precursors displaying featureless fracture surfaces without obvious voids or pre‐existing nanoscale porosity (Figure S10). In contrast, the micrographs of the materials obtained after selective etching revealed a well‐defined, interconnected nanoporous morphology across all formulations (Figure 5), confirming successful removal of the disulfide‐based macroCTA domains. The preservation of these nanostructures, without evidence of pore coalescence or structural collapse, further validates the choice of acetone as an optimal etching solvent.

These observations confirm that porosity arises strictly from the selective removal of macroCTA domains rather than from macroscopic defects or air bubbles introduced during the printing process. The transition from smooth precursor surfaces to well‐defined nanoporous networks is fully consistent with the microphase‐separated structure initially inferred from the SAXS analysis.

Statistical analysis of SEM images yielded average pore diameters of approximately 24, 32, 26, and 42 nm for NPT‐EHA, NPT‐ELp, NPT‐BA, and NPT‐EEA (Figures S11 and S12, Table S3), respectively, in excellent agreement with the SAXS‐derived domain spacings. This close correspondence confirms that pore formation proceeds via selective removal of the macroCTA domains, with the rigid crosslinked matrix preserving the original nanoscale architecture. Collectively, these results demonstrate that nanoporous morphology can be systematically tuned by varying macroCTA and matrix composition. Although nitrogen sorption (BET) analysis would provide the most direct quantification of specific surface area, reliable measurements could not be obtained for these materials: the glass transition temperatures of the crosslinked networks (53°C–81°C, determined by DMA; Figure S7) fall within or below the standard degassing temperature range (typically 60°C–120°C), such that thermal treatment prior to measurement induces softening and partial pore‐wall collapse, yielding irreproducible adsorption isotherms. This limitation is consistent with prior acrylate‐based PIMS studies employing comparable soft networks, which similarly did not report BET surface areas [15, 17].

The gravimetric analysis, SAXS, and SEM results confirm the selective removal of macroCTA domains from monolithic precursors, yielding nanoporous thermosets that retain both the characteristic PIMS length scale and macroscopic integrity. The preservation of domain spacing upon etching indicates that the crosslinked matrix provides sufficient mechanical rigidity to resist pore collapse or structural reorganization. Notably, this behavior persists across macroCTAs spanning a wide hydrophilicity range, from hydrophobic EHA to hydrophilic EEA, demonstrating broad compatibility of the etching protocol across diverse formulations. Importantly, macroCTA selection determines pore dimensions while matrix composition independently tunes pore‐wall chemistry, effectively decoupling nanoscale structure from surface functionality. This modularity makes the platform robust for designing nanoporous materials with tailored chemical environments and transport‐relevant architectures.

In addition, mechanical properties before and after etching were evaluated by tensile testing (Figures S13 and S14, Table S4). Prior to etching, all PIMS precursor thermosets exhibited good mechanical robustness, with Young's modulus ranging from 39.7 MPa to 93.0 MPa and ultimate tensile strengths up to 27.4 MPa. Following selective etching, the resulting nanoporous thermosets exhibited reduced stiffness and tensile strength, with Young's modulus decreasing to 5.9–71.1 MPa and ultimate tensile strengths to 1.0–13.5 MPa. This reduction is attributed to nanopore formation upon macroCTA removal, which diminishes the effective load‐bearing cross‐section and introduces localized stress concentration sites at pore walls. Notably, despite this reduction in stiffness, all NPTs retained macroscopic structural integrity without collapse or permanent deformation, confirming that the crosslinked acrylate matrix remains sufficiently robust to support the nanoporous architecture.

### 2.6 Integration with Additive Manufacturing

**FIGURE 6:**
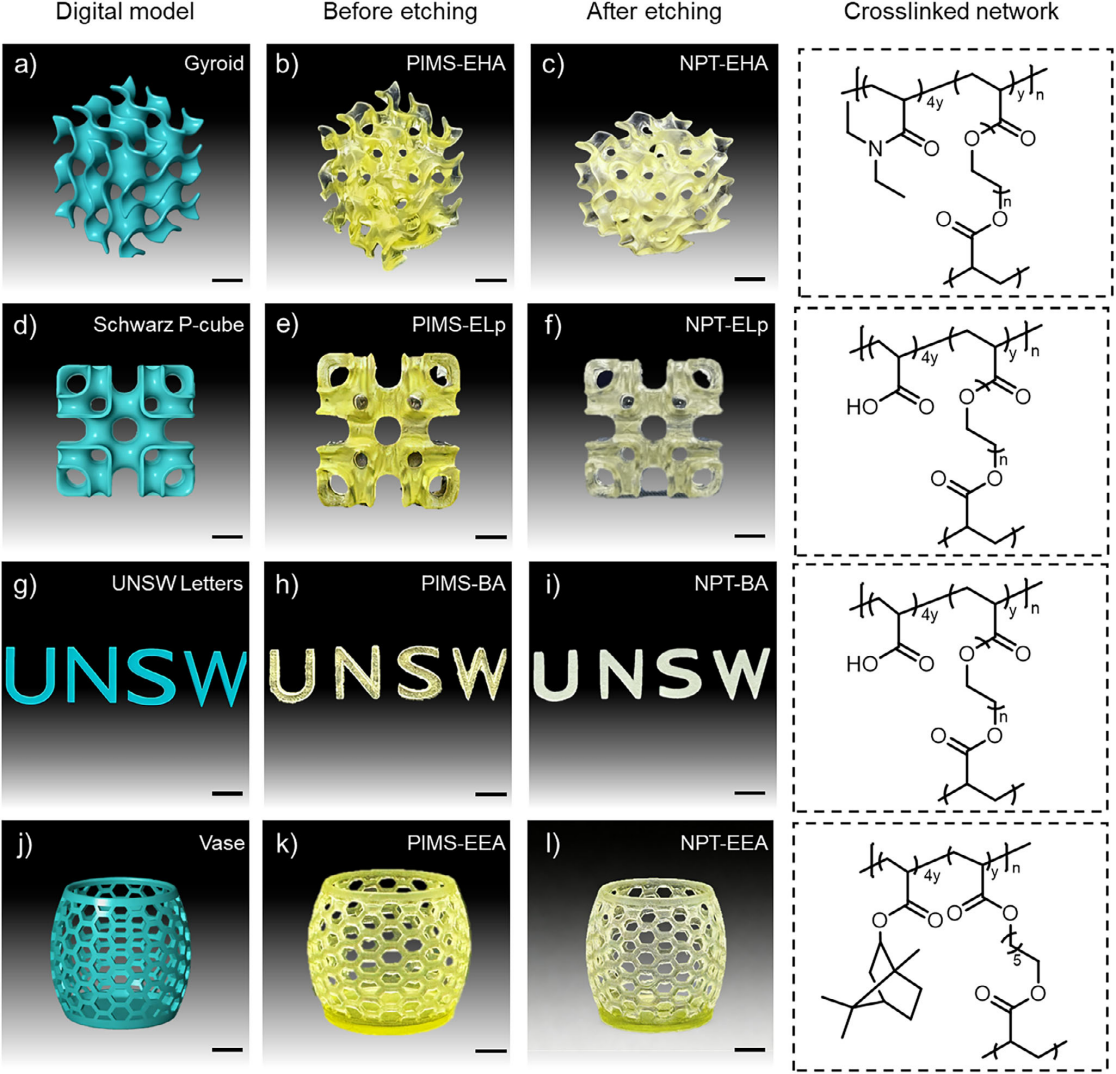
LCD 3D‐printed PIMS materials before and after etching. (a–c) Gyroid, (d–f) Schwarz P‐cube, (g–i) UNSW letters, (j–l) vase. For each: digital model (left), as‐printed precursor (middle), nanoporous thermoset after etching (right). Chemical structures of the crosslinked networks are shown on the far right. All architectures retained structural integrity after etching with visible translucency from nanoscale porosity. Scale bars: 5 mm.

To translate this modular pore‐forming chemistry into printed form factors, four representative architectures spanning distinct levels of geometric complexity were fabricated using a commercial LCD 3D printer (Figure 6). Examples included a gyroid structure (20 × 20 × 20 mm, wall thickness ∼0.8 mm), a Schwarz P‐cube (24 × 24 × 24 mm, wall thickness ∼1.0 mm), UNSW letters (height 10 mm), and a perforated vase (height 25 mm, wall thickness ∼0.6 mm). These geometries range from periodic minimal surfaces with high surface‐area‐to‐volume ratios to thin‐walled structures with complex curvature. The printing parameters were optimized through preliminary trials by systematically varying exposure time to ensure sufficient curing while maintaining feature resolution (Table S5). Under these optimized LCD conditions (layer thickness = 100 µm, per‐layer exposure time = 20 s, Z‐lift speed = 0.5 mm s^−1^, Z‐retract speed = 6 mm s^−1^), the printed structures closely matched their digital models across all tested geometries, confirming reliable cure kinetics and high‐fidelity reproduction of fine features. Notably, a single set of print parameters sufficed across all four PIMS formulations, underscoring the robustness and modularity of the resin platform.

The 3D‐printed materials were subsequently etched in 0.5 M DBU/acetone for 2 h at room temperature, following the same protocol established for bulk monoliths (Figure S15). All samples exhibited mass losses of 20–25 wt% (Table S6), comparable to both the theoretical macroCTA loading (30 wt%) and the values recorded for the thinner bulk specimens (26–29 wt%). This minor discrepancy in mass loss reflects an inherent trade‐off between quantitative macroCTA removal and structural preservation in thicker printed features. While extended etching facilitates greater mass loss, it also induces excessive swelling that can lead to fragmentation of the complex printed geometries. Consequently, an etching duration of 2 h was therefore selected as optimal, balancing substantial macroCTA removal with retention of macroscopic geometry and mechanical integrity.

Cross‐sectional SEM imaging (Figures S16–S18) revealed uniformly distributed nanopores throughout the bulk of the printed structures, with no evidence of pore size gradients, collapsed regions, or dense interfacial layers at interlayer positions. This confirms that DBU diffuses efficiently through the crosslinked network to access macroCTA domains across the full cross‐section, including within complex 3D‐printed geometries fabricated layer‐by‐layer. Comparable nanoporous morphologies were observed across all examined cross‐sectional regions, and no structural discontinuities or preferential etching at layer interfaces were detected (Figure S19). Collectively, these observations demonstrate that the selective etching process proceeded uniformly throughout the printed architecture and was not measurably affected by the inherent layer‐by‐layer nature of the LCD printing process, a finding of practical significance for the fabrication of geometrically complex nanoporous objects with isotropic pore morphology.

Although etching proceeded with retention of overall geometry and structural integrity, we observed a modest but measurable shrinkage of 6%–10% as quantified by image analysis (Table S7). The extent of shrinkage varied slightly across formulations, likely reflecting differences in crosslink density and network rigidity across the PIMS systems. More densely crosslinked networks are expected to better resist volumetric contraction upon macroCTA removal, retaining a greater fraction of the intended pore volume, whereas more compliant networks may undergo partial pore collapse and greater macroscopic shrinkage through viscoelastic relaxation of the matrix. As shown in the third row of Figure 6, the originally transparent prints became visibly translucent after etching. This optical transition is driven by the introduction of nanoscale porosity, with features smaller than the wavelengths of visible light, which increase refractive‐index differences that enhance light scattering at the pore‐matrix interfaces [12]. Despite slight dimensional shrinkage and reduced optical transparency, all printed objects retained their overall geometry and structural integrity following etching, including thin‐featured gyroid and vase architectures with wall thicknesses below 1 mm.

## 3 Conclusion

In summary, this work establishes a degradable PIMS platform that combines polymerization‐induced microphase separation with programmed disulfide cleavage to deliver nanoporous thermosets from readily printable photocurable resins. A library of LA‐ and ELp‐derived macroCTAs with systematically tunable hydrophilicity enables microphase separation across a broad formulation space, while preserving a built‐in chemical handle for post‐polymerization pore generation. Under mild basic conditions, DBU selectively cleaves and disrupts the disulfide‐containing macroCTA domains, removing the sacrificial phase and converting PIMS precursors into interconnected nanoporous networks with well‐defined pore diameters (24–42 nm). Importantly, the ability to tune macroCTA polarity decouples pore formation from formulation compatibility, while variation of the acrylate matrix chemistry provides an independent lever to tailor pore‐wall composition and functionality—establishing a modular route to nanostructured thermosets with application‐relevant surface chemistries. A key advance of this work is the direct integration of this chemistry with additive manufacturing. The PIMS formulations are photocurable and compatible with LCD 3D printing, enabling complex architectures to be fabricated first and then etched in a single post‐processing step to introduce nanoscale porosity throughout the printed object while preserving macroscopic fidelity and mechanical integrity, even in thin‐featured structures. Collectively, these results bridge a persistent gap between 3D‐printable form factors and programmable nanoscale structure, providing a general strategy for creating hierarchically structured polymers that combine geometric complexity with transport‐active porosity. More broadly, integrating cleavable disulfide macroCTAs with PIMS expands the accessible design space for nanoporous materials and should accelerate applications in separations, catalysis, and advanced manufacturing where both architecture and mass transport must be engineered simultaneously.

## Author Contributions

**Xueheng Dai**: investigation, validation, methodology, formal analysis, data curation, writing – original draft. **Kenny Lee**: investigation, writing – review and editing, formal analysis, data curation. **Yuan Xiu**: investigation, validation, formal analysis, data curation, writing – review and editing. **Nathaniel Corrigan**: investigation, formal analysis, writing – review and editing. **Kun Zhou**: investigation, validation, formal analysis, data curation, writing – review and editing. **Xichuan Li**: writing – review and editing, investigation, formal analysis. **Christopher M. Bates**: investigation, writing – review and editing, supervision. **Craig J. Hawker**: investigation, conceptualization, writing – review and editing, resources, supervision. **Cyrille Boyer**: conceptualization, funding acquisition, writing – review and editing, writing – original draft, supervision, resources, project administration.

## Conflicts of Interest

The authors declare no conflicts of interest.

## Supporting information




**Supporting File**: Experimental details, Scheme S1, Tables S1–S7, and Figures S1–S19 are available in Supporting Information.

## Data Availability

The data that support the findings of this study are available from the corresponding author upon reasonable request.
