# Supplementary material for: 3D‐Printable Nanoporous Thermosets via Disulfide‐Based Polymerization‐Induced Microphase Separation
Source: Angew Chem Int Ed Engl. 2026 Jun 13;65(33):e8227484. doi: 10.1002/anie.8227484 (PMC13452562; doi:10.1002/anie.8227484)
Supplement: Supplementary file 1 [file ANIE-65-e8227484-s001.pdf]

Supporting Information

3D-Printable Nanoporous Thermosets via Disulfide-Based Polymerization-Induced Microphase Separation

Xueheng Dai,[a] Kenny Lee,[a,b] Yuan Xiu,[a] Nathaniel Corrigan,[c] Kun Zhou,[a] Xichuan Li,[a] Christopher M. Bates,*[b] Craig J. Hawker,*[b] and Cyrille Boyer*[a]

[a]Cluster for Advanced Macromolecular Design (CAMD) and Australian Centre for Nanomedicine (ACN), School of Chemical Engineering, University of New South Wales, Sydney, NSW 2052, Australia.

[b]Materials Research Laboratory and Materials Department, University of California, Santa Barbara, Santa Barbara, California 93106, United States.

[c]Centre for Advanced Manufacturing Technology (CfAMT), School of Engineering, Western Sydney University, Sydney, NSW 2747, Australia.

Correspondence: Christopher M. Bates (cbates@ucsb.edu), Craig J. Hawker (hawker@ucsb.edu), and Cyrille Boyer (cboyer@unsw.edu.au)

Materials and Supplementary Methods

Materials

α-Lipoic acid (LA, ≥ 99.49%) and 1-(3-dimethylaminopropyl)-3-ethylcarbodiimide hydrochloride (EDC-HCl, ≥ 99 %) were purchased from Ambeed and used as received. 4- Dimethylaminopyridine (DMAP, ≥ 98 %), dichloromethane (DCM, ≥ 99.5 %), ethanol (EtOH, ≥ 99.9 %), methanol (MeOH, ≥ 99.9%), acetone (≥ 99.95 %), 1,8-diazabicyclo(5.4.0)undec-7-ene (DBU, ≥ 99.0 %), diphenyl(2,4,6-trimethylbenzoyl)phosphine oxide (TPO, > 97%), chloroform-d ($CDCl_3$, 99.8 atom % with 1 % v/v tetramethylsilane), acrylic acid (AA, ≥ 99.0%), 1,6-hexanediol diacrylate (HDDA, ≥ 80%), poly(ethylene glycol) diacrylate (PEGDA, M_n = 250 g mol^{-1}, ≥ 92.0%), and 2,2'-azobis(2-methylpropionitrile) solution (AIBN, 0.2 M in toluene) were purchased from Sigma Aldrich and used as received. 2-ethylhexyl acrylate (EHA, ≥ 98.0%), isobornyl acrylate (IBoA, ≥ 99%), and *n*-butyl acrylate (BA, ≥ 99%) were purchased from Sigma Aldrich and passed through a basic alumina column (100-200 mesh, Fluka) to remove inhibitors. *N,N*-diethylacrylamide (DEAm, ≥ 98.0%) and 2-(2-ethoxyethoxy)ethyl acrylate (EEA, ≥ 98.0%) were purchased from Tokyo Chemical Industry and passed through alumina column to remove inhibitors. 4-Cyano-4-(dodecylthio)carbonothioyl)thio)pentanoic acid (CDTPA, ≥ 97%) was purchased from Boron Molecular and used as received. Sodium chloride (NaCl, Chem-Supply, ≥ 99%), sodium hydrogen carbonate ($NaHCO_3$, ≥ 99.7), magnesium sulfate ($MgSO_4$, ≥ 62%), hexane (≥ 99%), and diethyl ether (≥ 99.5%) were purchased from Chem-Supply and used as received. (Trimethylsilyl)diazomethane was purchased from Aladdin Scientific Corporation and used as received. Aqueous hydrogen chloride (HCl, 32 %), tetrahydrofuran (THF, ≥ 99.8 %), and 1,4 dioxane (≥ 99.8 %) were purchased from RCl Labscan and used as received.

Supplementary Methods

<u>Synthesis of ethyl lipoate (ELp)</u>

Ethyl lipoate (ELp) was synthesized via EDC coupling as follows: initially, DCM was dried over anhydrous $MgSO_4$ to remove trace water. 30.0 g of LA (0.15 mol, 1 equiv), 6.7 g EtOH (0.15 mol, 1 equiv), and 3.5 g DMAP (0.03 mol, 0.2 equiv) were dissolved in 50 g dry DCM in a 500 mL round bottom flask containing a magnetic stirrer bar. After complete dissolution of reactants, the round bottom flask was then cooled to 0 °C in an ice bath and allowed to cool for 60 min. Separately, 33.4 g EDC-HCl (0.17 mol, 1.2 equiv) was dissolved in 120 g dry DCM. The EDC-HCl in DCM solution was then added dropwise using a syringe to the 500 mL round bottom flask containing LA, EtOH and

DMAP under magnetic stirring. After all reactants had been combined, the reaction setup was covered in aluminium foil and allowed to react for 17 h. After reacting, the crude mixture was then washed with 1 M HCl in water (2 × 100 g), saturated $NaHCO_3$ (2 × 100 g) and brine (2 × 100 g NaCl). Here, the base wash using saturated $NaHCO_3$ is crucial to remove trace unreacted LA which may catalyse cationic polymerization/depolymerization of resulting polymers. The extracted mixture was then dried overnight over anhydrous $MgSO_4$. The DCM was then removed using a rotary evaporator, and the product was stored in a desiccator for at least 24 h before use.

Procedure for macroCTA synthesis

For the synthesis of P(LA-*co*-EHA)-macroCTA: LA (39 equiv, 10.38 g, 50.31 mmol), EHA (91 equiv, 21.63 g, 117.39 mmol), CDTPA (1 equiv, 0.52 g, 1.29 mmol), and AIBN (0.2 equiv, 0.2 M solution in toluene, 1.29 mL, 0.26 mmol) were dissolved in 1,4-dioxane (76 mL). The mixture was deoxygenated by purging with argon for 60 min and then heated at 70 °C for 18 h. The polymerization was quenched by cooling in an ice bath for 20 min and exposing to air. The crude polymer solution was precipitated in excess cold methanol and isolated by centrifugation. Fresh methanol was added to the polymer residue, and the mixture was vortexed and centrifuged. This washing-centrifugation cycle was repeated three times. The isolated polymer was then dissolved in DCM (50 mL), concentrated using a rotary evaporator, and dried under vacuum at 40 °C overnight.

Using the same protocol, P(ELp-*co*-BA)-macroCTA was successfully synthesized. Specifically, P(LA-*co*-BA)-macroCTA and P(LA-*co*-EEA)-macroCTA were copolymerized following an identical procedure, except that the crude solution was precipitated in excess mixed solvent of diethyl ether and hexane (50/50 v/v) and centrifuged at 9500 rpm for 5 min at 10 °C, followed by rotary evaporation and vacuum drying.

Photocurable resin preparation

Photocurable PIMS resins were prepared by mixing the macroCTA, vinyl monomer, multifunctional acrylate crosslinker, and photoinitiator at room temperature. In all formulations, the macroCTA loading was fixed at 30 wt%, the molar ratio of vinyl monomer to crosslinker was maintained at 4:1, and diphenyl(2,4,6-trimethylbenzoyl)phosphine oxide (TPO) was added at 0.5 wt% relative to the total resin mass.

The macroCTA was first dissolved in the vinyl monomer by sonication and vortex mixing at room temperature for 10-15 min. The crosslinker and TPO were then added sequentially, and the mixture was vortexed and sonicated for an additional 30 min in the dark to prevent premature

photopolymerization. All formulations yielded transparent, homogeneous resins that were used immediately or stored in 4 mL vials at 4 °C.

Photopolymerization and bulk monolith fabrication

Bulk PIMS precursors: the photocurable resins were used to fabricate bulk monoliths (8 × 8 × 2 mm) using a commercial LCD 3D printer (Anycubic Photon S) equipped with a 405 nm LED array (I_0 = 3.5 mW cm^{-2}). The resin was poured into the resin vat, and samples of prism models (8 × 8 × 2 mm) were 3D printed at the thickness of 20×100 µm layers. After printing, the samples were rinsed with ethanol to remove uncured resin and dried in air at room temperature. The resulting PIMS precursor monoliths were transparent to slightly yellow in colour and mechanically robust, suitable for subsequent etching and characterization.

SAXS sample preparation

Square prism samples (6 × 6 × 0.2 mm) were prepared using a commercial LCD 3D printing on a commercial printer (Anycubic Photon S) equipped with a 405 nm LED array (I_0 = 3.5 mW cm^{-2}). SAXS sample dimensions were selected to ensure consistent dimensions and facilitate mounting in the SAXS sample holder.

LCD 3D printing

3D architectures were fabricated using a commercial LCD 3D printer (Anycubic Photon S) equipped with a 405 nm LED array (I_0 = 3.5 mW cm^{-2}). The building stage was calibrated according to the manufacturer's instructions, and the resin vat was cleaned with ethanol before use. Digital 3D models (gyroid, Schwarz P-surface, UNSW lettering, and vase geometries) were designed in Anycubic Photon Workshop and exported as STL files.

Printing optimization: Printing parameters were optimized through preliminary trials in which exposure time (10–40 s) was systematically varied to balance print speed and degree of cure while preserving feature resolution of model objects (see **Table S5**). Throughout these trials, resin compositions were held constant at 30 wt% macroCTA, 0.5 wt% TPO photoinitiator, and a vinyl monomer-to-crosslinker molar ratio of 4:1, with layer thickness and light intensity fixed at 100 µm and 3.5 mW cm^{-2}, respectively. The optimized conditions were determined to be per-layer exposure time of 20 s, and bottom-layer exposure time of 40 s.

3D printing: The photocurable PIMS resin (5-10 g) was poured into the resin vat, and the print job was initiated. Printing was carried out in a few hours depending on object height (with a Z lift speed

of 0.5 mm s^{-1} and a Z retract speed of 6 mm s^{-1}). After printing, the build stage was raised, and the printed object was carefully removed using a plastic spatula. Printed objects were rinsed with ethanol to remove uncured resin from the surface, then allowed to dry in air at room temperature for 1 h. Additional 405 nm post-curing was performed for 15 min to ensure sufficient vinyl monomers conversion. The printed objects were stored in the dark at room temperature until etching.

Degradation of macroCTAs using DBU/acetone

The macroCTAs (30 mg) were dissolved in 1 mL 0.5 M 1,8-diazabicycloundec-7-ene (DBU)/acetone solution in a glass vial and stirred at room temperature. After 2 h degradation, 2 drops were withdrawn and quenched with 2 drops of distilled water and analysed by THF SEC to monitor the decrease in molar mass over time.

Selective etching of 3D printed materials in DBU/acetone

Solvent screening: PIMS-EHA monoliths (~30 mg each) were etched in different solvents (toluene, acetone, methanol) containing 0.5 M DBU for 2 h at room temperature. After washing with the respective pure solvent (3 × 20 mL) and drying, mass losses and structural integrity were evaluated. Only acetone provided a balance between efficient macroCTA degradation and structural preservation. A stock solution of 0.5M DBU/acetone was prepared fresh for each etching experiment.

Etching for bulk monoliths: PIMS precursor monoliths (~30 mg) were placed in 20 mL glass vials and covered with the DBU/acetone solution (10 mL). The vials were capped and placed without stirring at room temperature for 2 h.

Etching for 3D-printed architectures: 3D-printed objects were placed in appropriately sized glass containers and covered with 0.5 M DBU/acetone solution. For large objects (e.g., gyroid, vase), 150 mL of etching solution was used. Gentle agitation was provided by occasional manual swirling (every 30 min) to ensure uniform access of the etching solution.

After 2 h, the samples were removed from the etching solution using tweezers or a spatula and immediately immersed in fresh acetone for washing for 10 min. The washing procedure was repeated three times (3 × 50 mL, 10 min each) with fresh acetone to remove residual DBU and soluble degradation products. The samples were then transferred to a vacuum oven and dried at 40 °C under reduced pressure (< 10 mbar) for overnight to constant mass.

Characterization

Nuclear magnetic resonance (NMR)

^1H NMR spectra were recorded on a Bruker Avance III 400 MHz spectrometer at 25 °C. Chemical shifts (δ) are reported in ppm relative to residual portion solvent in $CDCl_3$ (7.26 ppm).

Size-exclusion chromatography (SEC)

Analysis was performed using tetrahydrofuran (THF) as the eluent. The SEC system was a Shimadzu modular system comprising an auto injector, oven at 40 °C, a Phenomenex 5.0 µm bead-size guard column (50 × 7.5 mm) followed by two Agilent MIXED C columns (PLgel 5µm), and a Shimadzu RID-10A refractive-index detector. The system was calibrated with Agilent narrow molecular weight distribution poly(methyl methacrylate) (PMMA) standards with molecular weights (M_n) ranging from 545 to 2,210,000 g mol^{-1}. Prior to SEC analysis, all lipoic acid (LA)-based macroCTAs were methylated using (trimethylsilyl)diazomethane to mask the carboxylic acid groups and minimize undesired interactions with the SEC columns.

Small-angle X-ray scattering (SAXS)

SAXS measurements were conducted on an Anton Paar SAXSPoint 2.0 system with a Cu K_α (λ = 0.154 nm) microfocus X-ray source and Dectris Eiger 1M detector. Data was acquired at room temperature, under vacuum for 5 min from the sample chamber at a sample-to-detector distance of 0.575 m. Objects were printed at the thickness of 0.2 mm. Data was reduced to 1D by radial averaging the 2D detector after converting pixel positions to $q = (4\pi/\lambda)\sin\theta$, where 2θ is the scattering angle. The domain spacing could be obtained using **Equation (Eq. S1) (Supporting Information):**

$$d_{\mathrm{SAXS}} = \frac{2\pi}{q*} \quad \textbf{(Eq. S1)}$$

Scanning electron microscopy (SEM)

All samples were prepared on a stub with conductive tape and coated with platinum coating with 1 nm thickness by virtue of a Leica ACE600 sputter coater. SEM images were acquired using a field-emission NanoSEM 230 or 450 instrument equipped with a 3 kV accelerating voltage and a secondary electron detector.

Gravimetric analysis

Samples were weighed before etching (m_{ini}) and after drying (m_{fin}) using an analytical balance. Mass loss was calculated as **Equation (Eq. S2) (Supporting Information)**:

$$\text{Mass loss (wt\%)} = \frac{m_{ini} - m_{fin}}{m_{ini}} \times 100\% \qquad \textbf{(Eq. S2)}$$

Dynamic mechanical analysis (DMA)

DMA was performed using a single cantilever bending test. The sample dimensions were 30 mm (length) × 8 mm (width) × 2 mm (thickness). The analysis was performed using a TA instruments Q800 dynamic mechanical analyser which was equipped with a TA instruments liquid nitrogen gas cooling accessory (GCA) for temperature control. Initially, the 3D printed sample was measured using digital calipees and placed into the single cantilever clamp. The clamp was then tightened using a torque wrench with a force of 5 in lb. All experiments were conducted using the following method: equilibration at -70 °C, isothermal for 3 min, temperature ramped to 100 °C at a rate of 2 °C/min, constant frequency of 1 Hz and displacement of 20 μm. Glass transition temperature (T_g) was determined using the temperature at the peak of the tan δ curve.

Attempted nitrogen sorption (BET) analysis

Nitrogen sorption measurements were attempted on etched NPT samples (after etching) using a Micromeritics instrument with standard degassing conditions. Reliable and reproducible adsorption isotherms could not be obtained across all formulations. This is attributed to the low glass transition temperatures of the crosslinked acrylate networks (T_g = 53–81 °C, determined by DMA), which fall within or below the standard degassing temperature range (60–120 °C). Thermal treatment at these temperatures induces softening and partial collapse of the nanopore walls, yielding irreproducible BET surface area values. Accordingly, specific surface area data are not reported. Porosity is instead evidenced by the convergent characterization approach described in the main text (gravimetric analysis, SAXS, SEM, and DMA).

Tensile testing

The mechanical tensile stress tests were performed using a Mark–10 ESM303 with a 1 kN force gauge model M5–200. The speed of testing was 2.1 mm/min. All tensile results were performed in at least quintuplicate. The tensile stress was calculated from the applied force divided by the initial cross-sectional area of the gage section. The strain was determined as the change in gage length relative to the original specimen gage length, expressed as a percent.

Additional Data

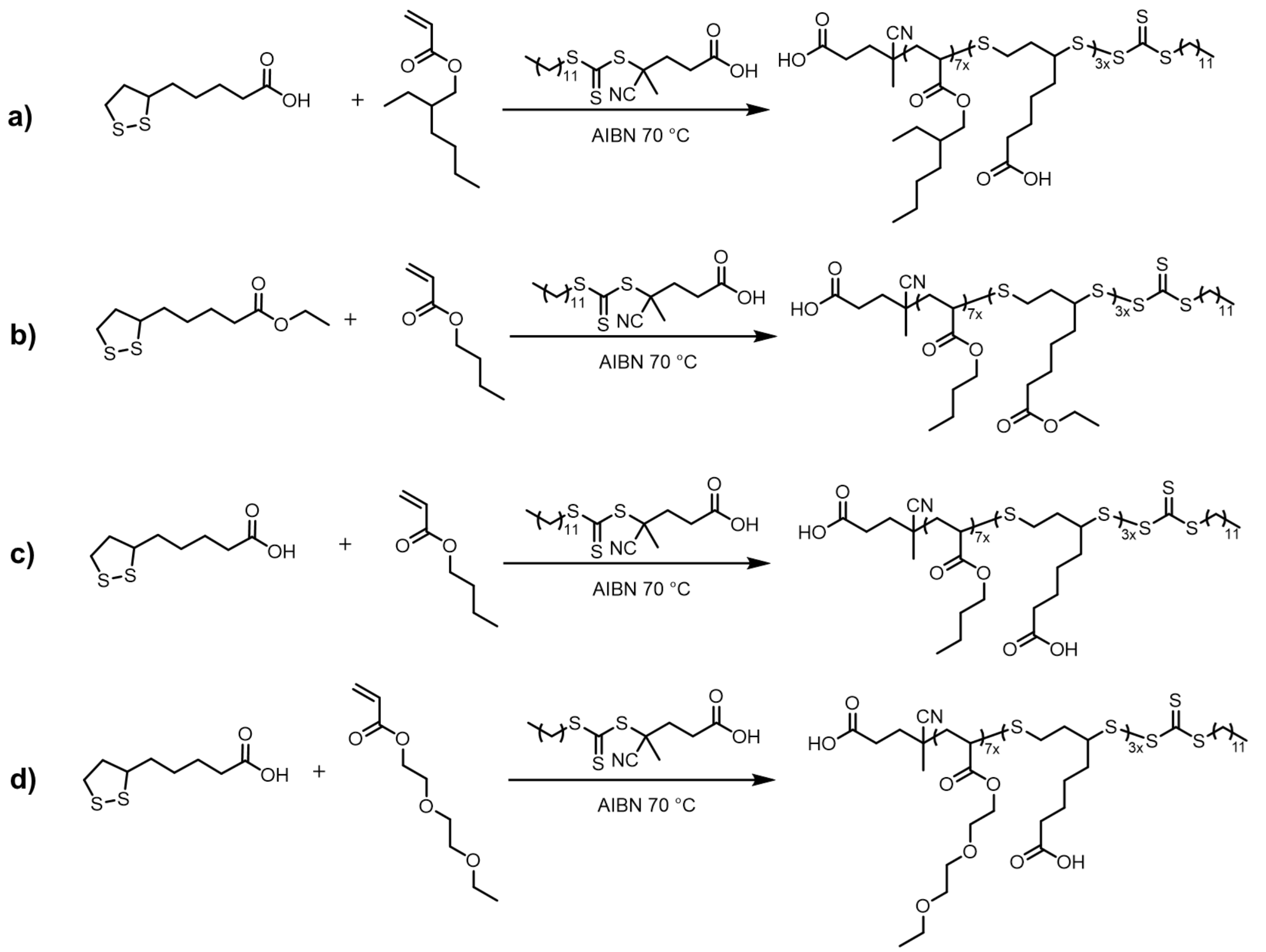

Scheme S1. Synthesis of macroCTAs using RAFT copolymerization. Synthesis of a) P(LA-*co*-EHA)-macroCTA; b) P(ELp-*co*-BA)-macroCTA; c) P(LA-*co*-BA)-macroCTA; d) P(LA-*co*-EEA)-macroCTA.

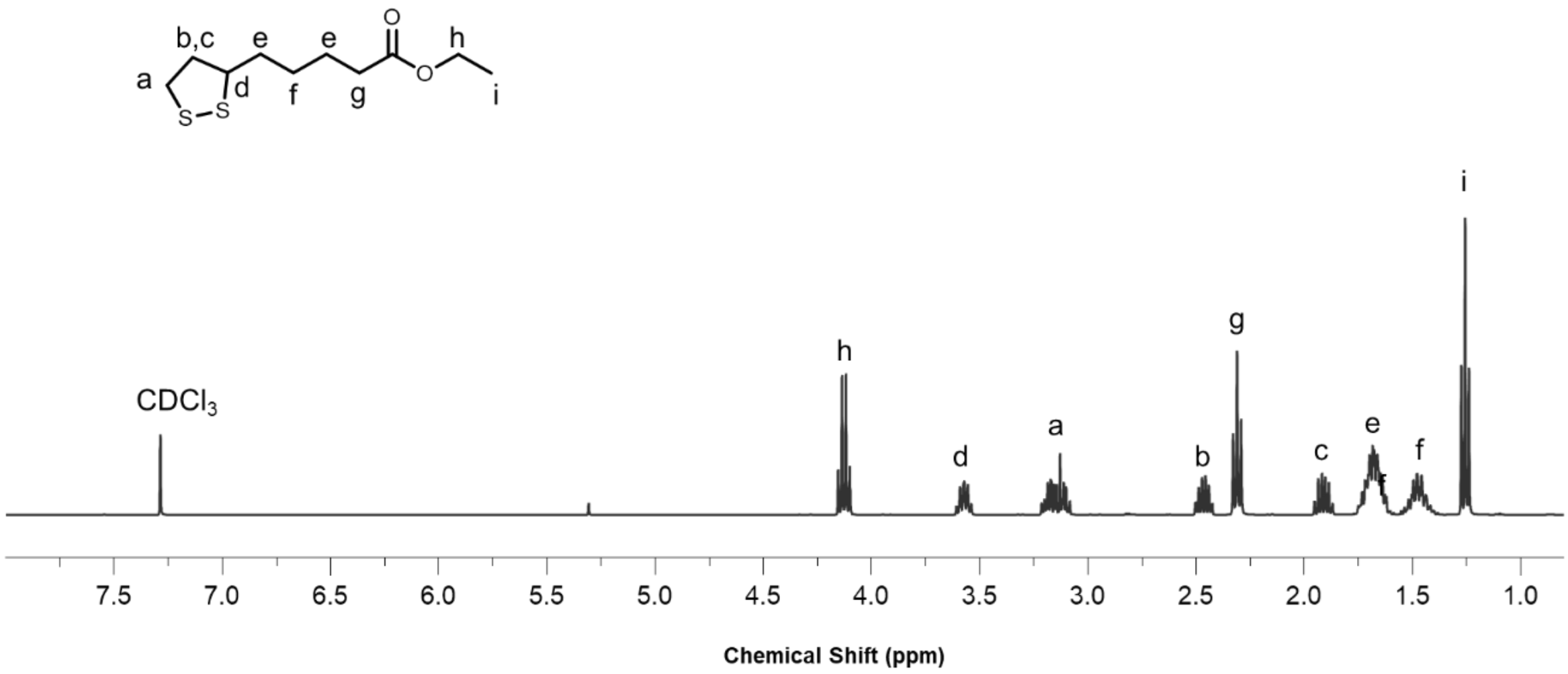

Figure S1. ^{1}H NMR spectrum of synthesized ethyl lipoate (ELp). Spectrum recorded in $CDCl_3$, 400 MHz, 298 K.

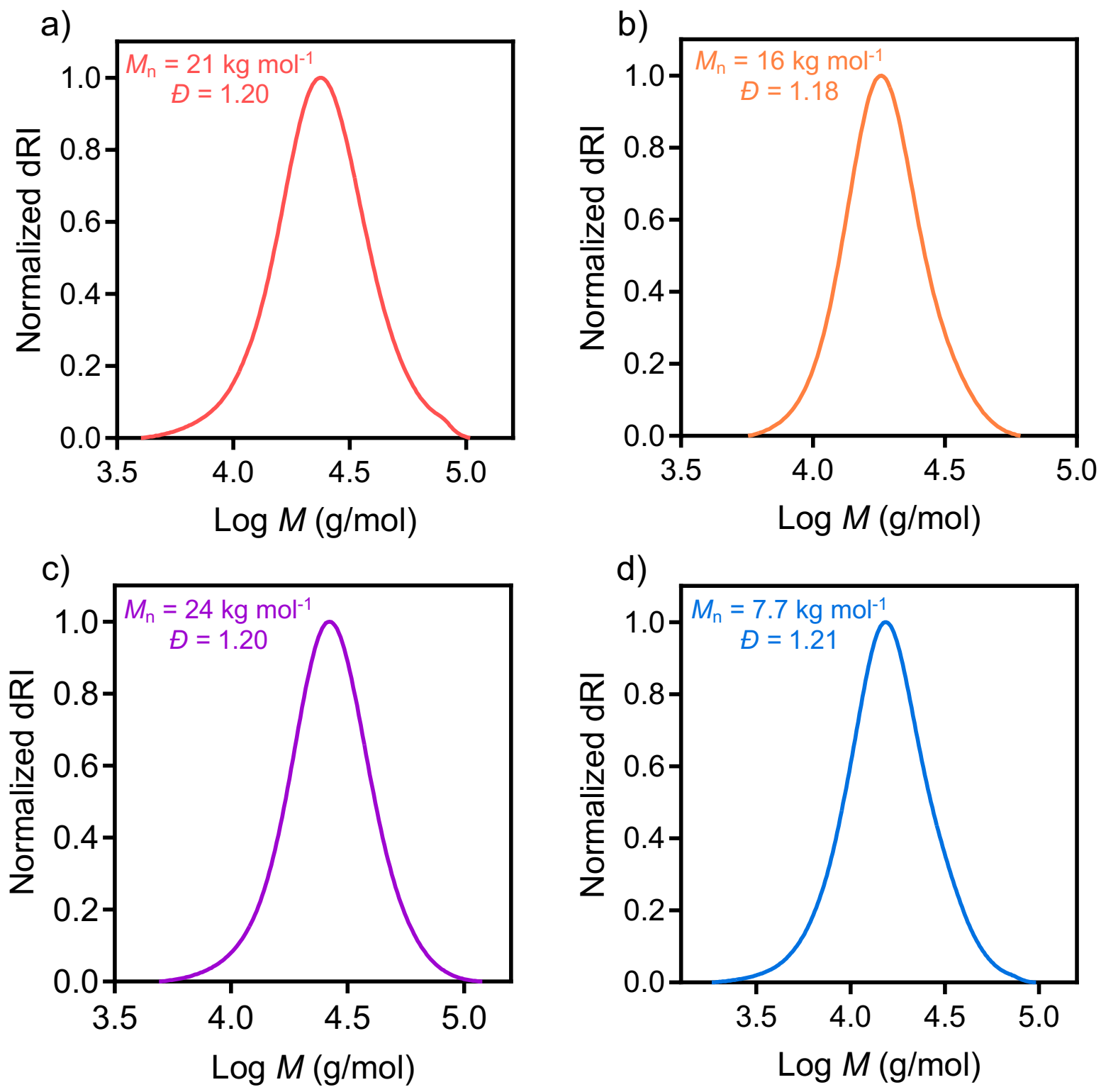

Figure S2. SEC traces of disulfide-containing macroCTAs. (a) P(LA-*co*-EHA)-macroCTA, (b) P(ELp-*co*-BA)-macroCTA, (c) P(LA-*co*-BA)-macroCTA, and (d) P(LA-*co*-EEA)-macroCTA. Molar mass and dispersity are indicated on each trace. All samples show monomodal distributions with $Đ < 1.3$. Note: obtained by THF SEC using PMMA calibration.

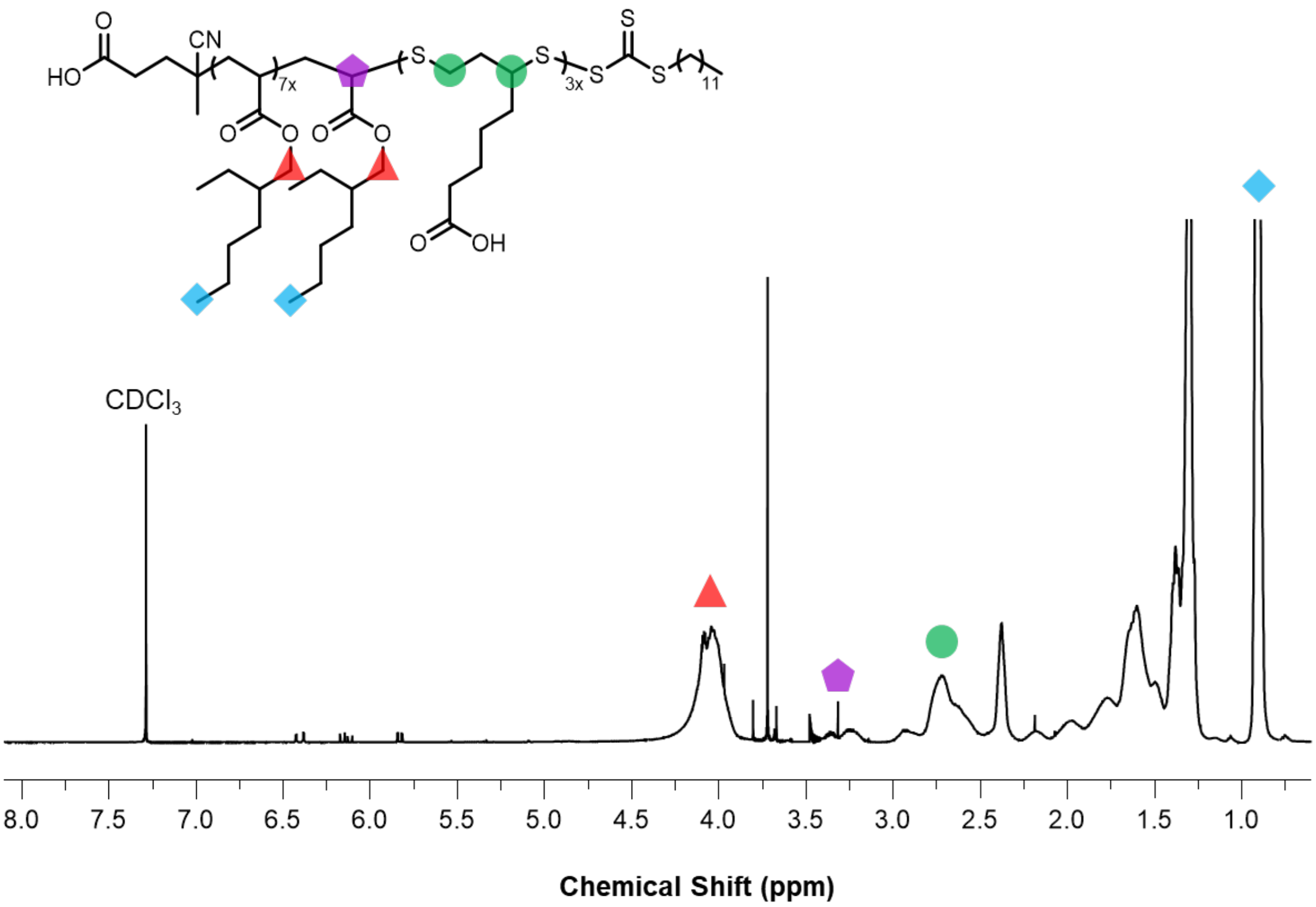

Figure S3. ^{1}H NMR spectrum of purified P(LA-*co*-EHA)-macroCTA. Spectrum recorded in $CDCl_3$, 400 MHz, 298 K.

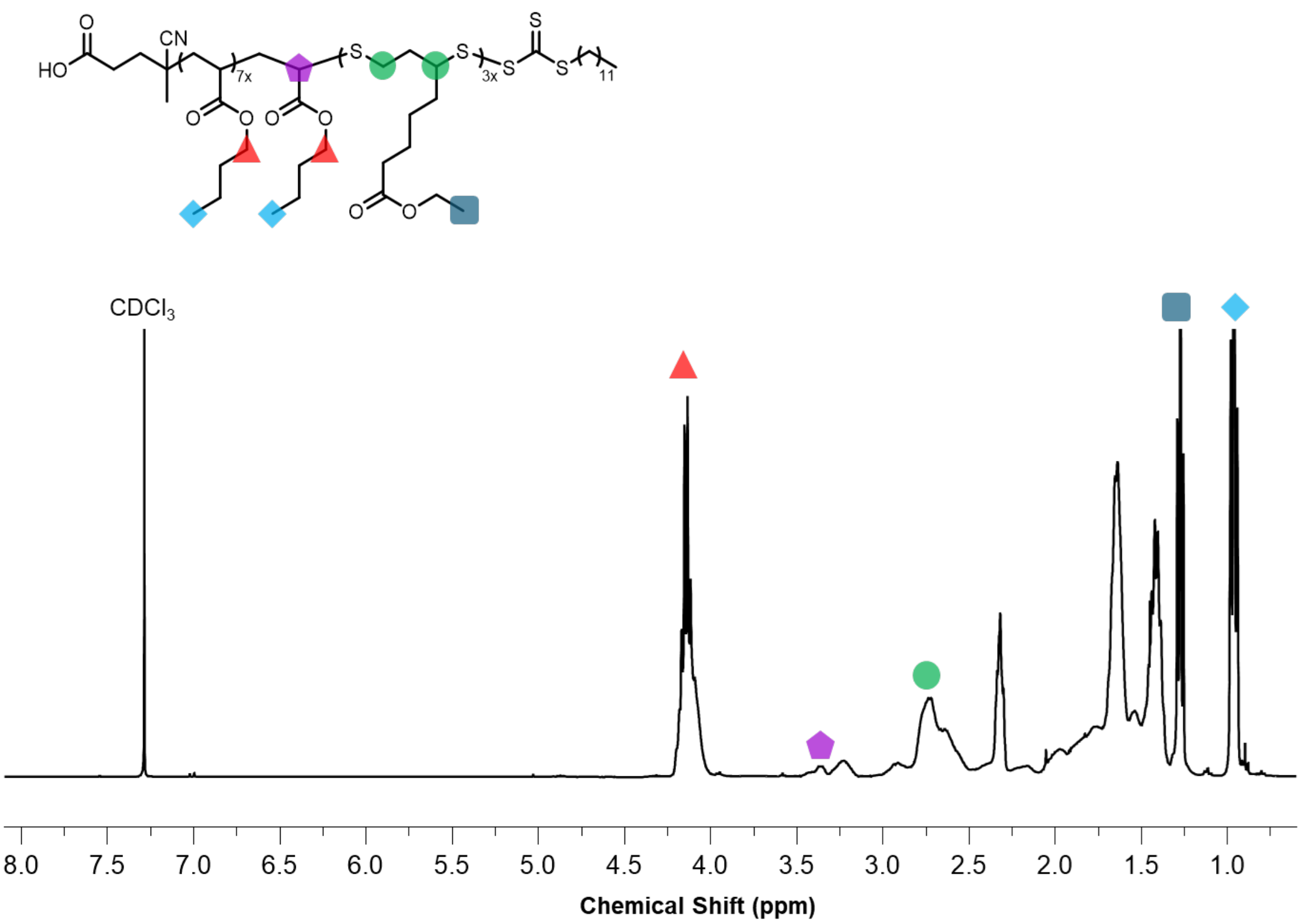

Figure S4. ^{1}H NMR spectrum of purified P(ELp-*co*-BA)-macroCTA. Spectrum recorded in $CDCl_3$, 400 MHz, 298 K.

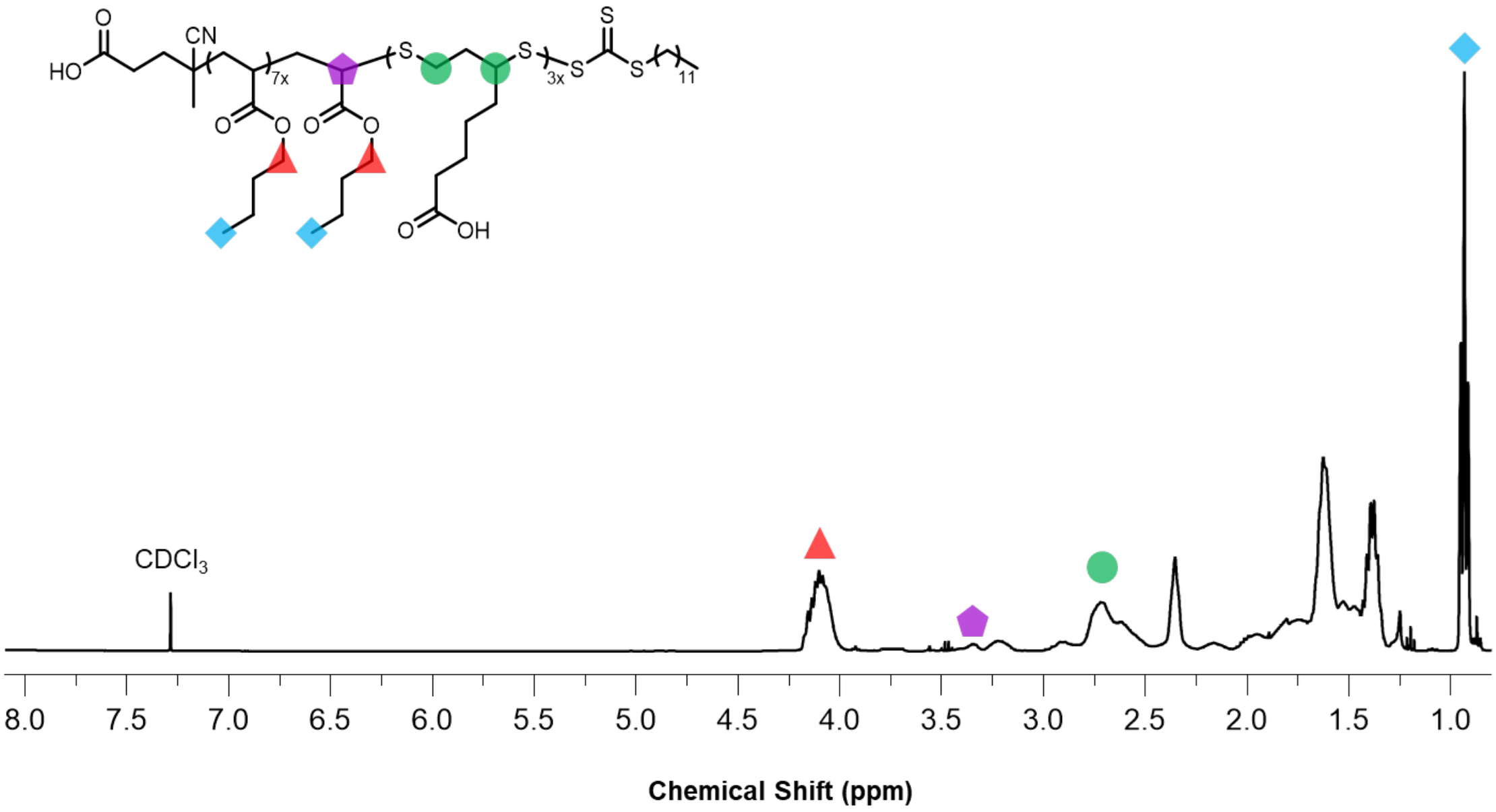

Figure S5. ^{1}H NMR spectrum of purified P(LA-*co*-BA)-macroCTA. Spectrum recorded in $CDCl_3$, 400 MHz, 298 K.

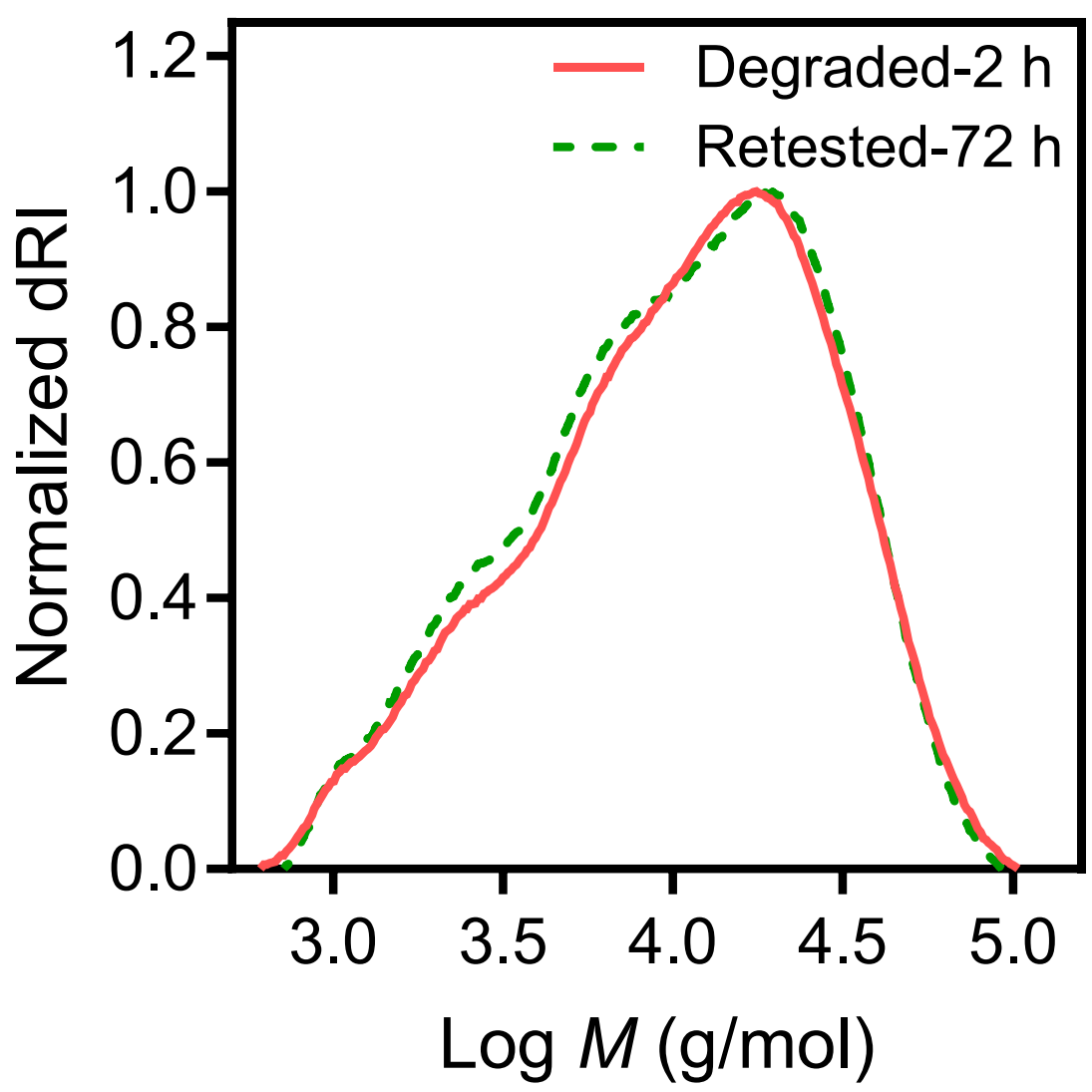

Figure S6. SEC traces of effective quenching of DBU-catalyzed degradation in P(ELp-*co*-BA)-macroCTA. After the 2 h reaction, the degradation was immediately quenched (orange solid line), and the sample was re-tested after 72 h (green dash line), the molecular weight distribution remained unchanged after quenching. Note: obtained by THF SEC using PMMA calibration.

Table S1. Compositions of photocurable resins for PIMS precursors. All formulations contain 30 wt% loading of macroCTA and 0.5 wt% TPO relative to the total resin mass. The remaining fraction consists of vinyl monomer and crosslinker at a monomer-to-crosslinker molar ratio of 4:1.

Entry	MacroCTA	Monomer	Monomer (wt%)	Crosslinker	Crosslinker (wt%)
1	P(LA-*co*-EHA)-macroCTA	DEAm	46.6	PEGDA	22.9
2	P(ELp-*co*-BA)-macroCTA	AA	37.3	PEGDA	32.2
3	P(LA-*co*-BA)-macroCTA	AA	37.3	PEGDA	32.2
4	P(LA-*co*-EEA)-macroCTA	IBoA	54.7	HDDA	14.8

Note: the exact masses may be adjusted to achieve the desired total volume while maintaining the specified weight percentages.

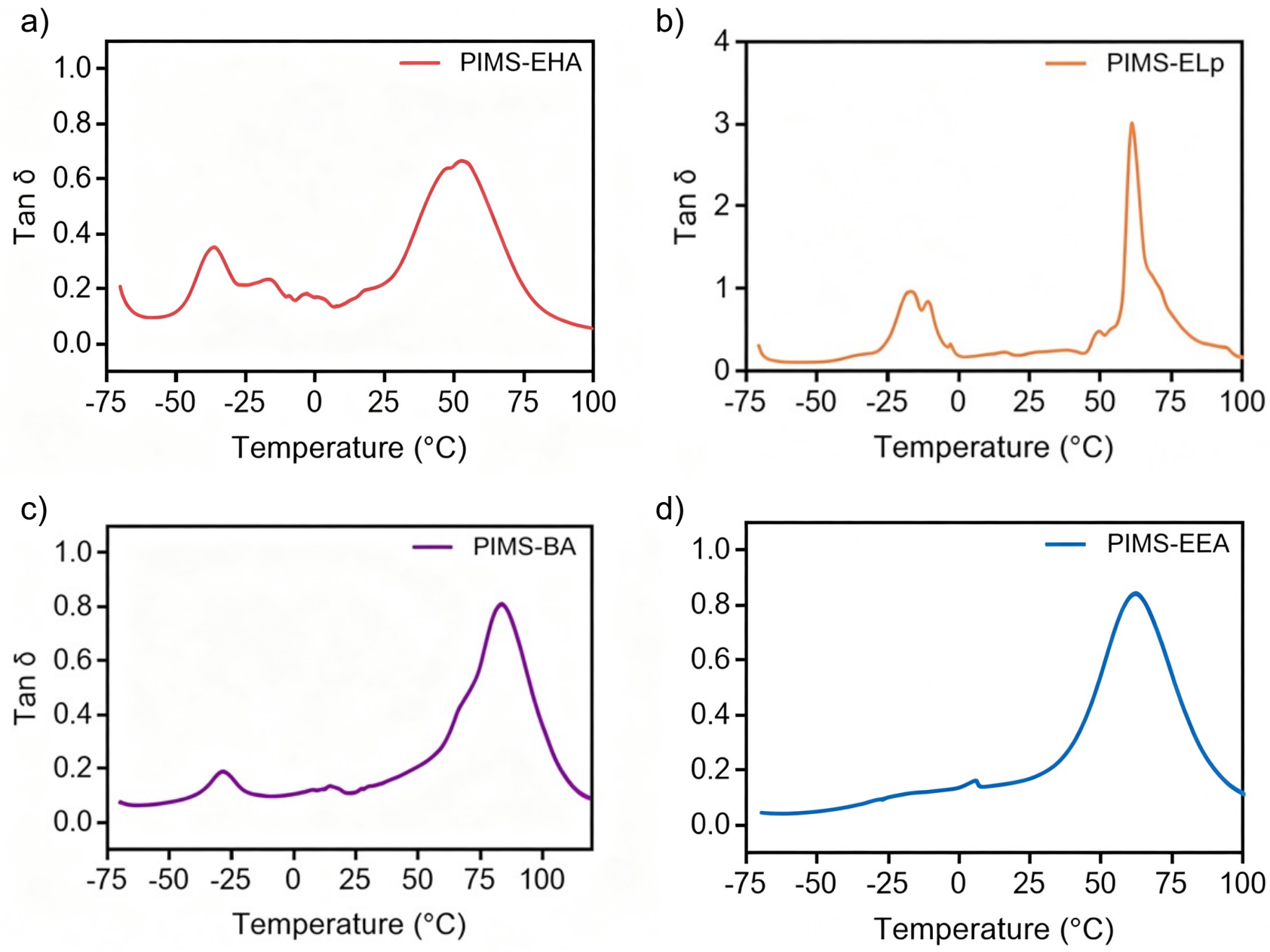

Figure S7. Tan δ curves as a function of temperature for 3D-printed PIMS precursor thermosets obtained by dynamic mechanical analysis (DMA). a) PIMS-EHA, b) PIMS-ELp, c) PIMS-BA, and d) PIMS-EEA. Two distinct transitions are observed in each profile, corresponding to the glass transitions of the macroCTA-rich phase (lower temperature) and the crosslinked matrix (higher temperature), consistent with microphase-separated morphologies evidenced by SAXS.

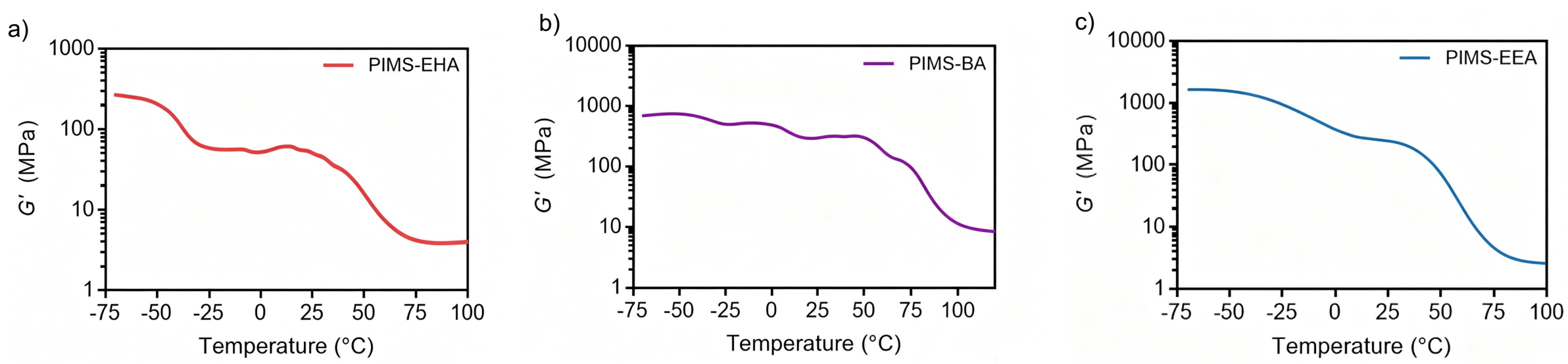

Figure S8. Storage modulus (*E*') as a function of temperature for 3D-printed PIMS precursor thermosets obtained by dynamic mechanical analysis (DMA), measured over the temperature range of −70 to 120 °C. a) PIMS-EHA, b) PIMS-BA, and c) PIMS-EEA. The plateau storage modulus in the rubbery region reflects the crosslink density of each network, while the onset of the drop in *E*' is consistent with the glass transition temperatures identified from the corresponding tan δ profiles (**Figure S7**).

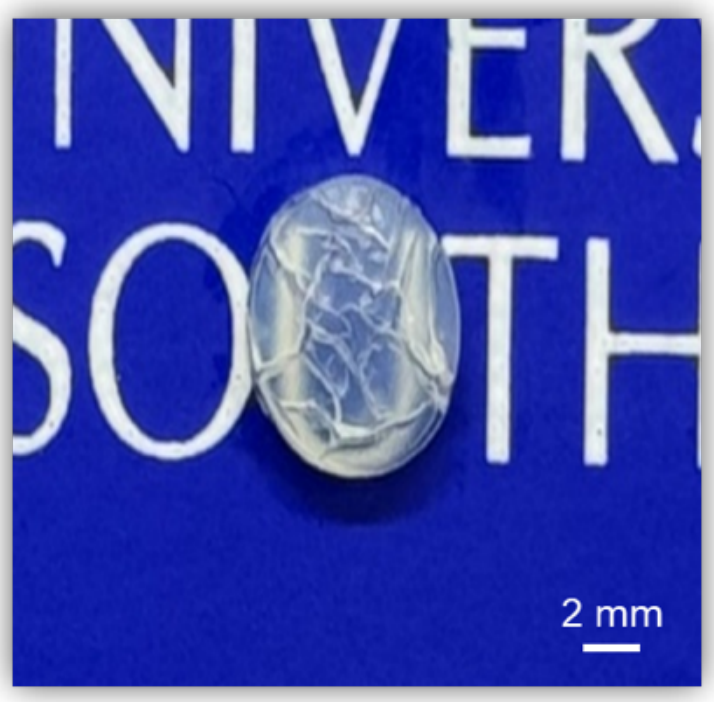

Figure S9. The photograph of etched PIMS-EHA monolith obtained by selective removal of P(LA-*co*-EHA)-macroCTA in 0.5 M DBU/methanol solution for 2 h at room temperature, demonstrating macrostructural degradation due to excessive swelling in methanol.

Table S2. Gravimetric mass loss of PIMS monoliths after selective etching via 0.5 M DBU in acetone.

Entry	Sample	Theoretical mass loss (wt%)	Experimental mass loss (wt%)
1	PIMS-EHA	30	26.2 ± 1.1
2	PIMS-ELp	30	28.3 ± 1.2
3	PIMS-BA	30	27.5 ± 0.8
4	PIMS-EEA	30	28.6 ± 0.6

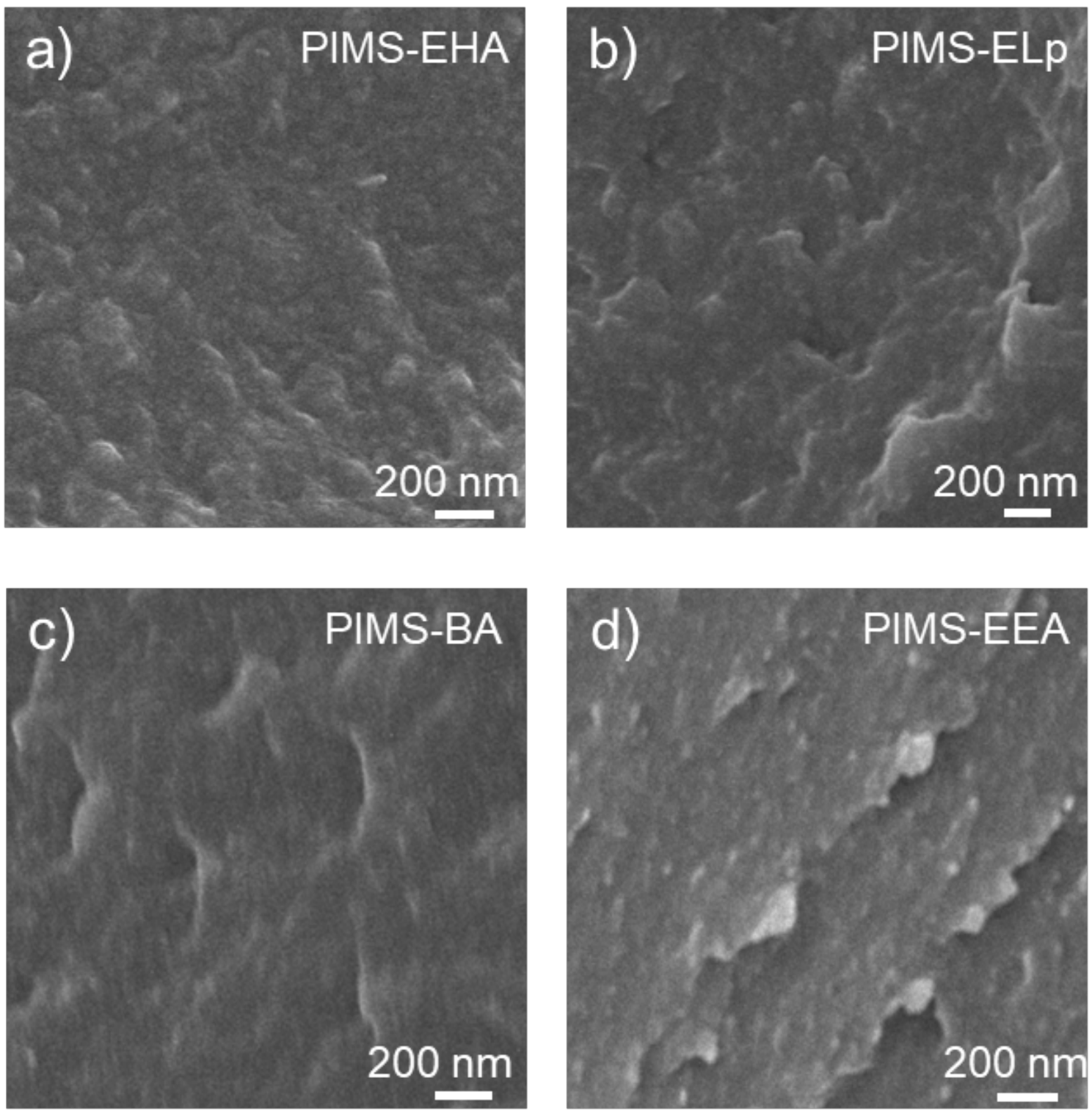

Figure S10. Cross-sectional SEM images of PIMS monoliths before etching. a) PIMS-EHA; b) PIMS-ELp; c) PIMS-BA; and d) PIMS-EEA. Note: samples were coated with thin layer of Pt (~1 nm).

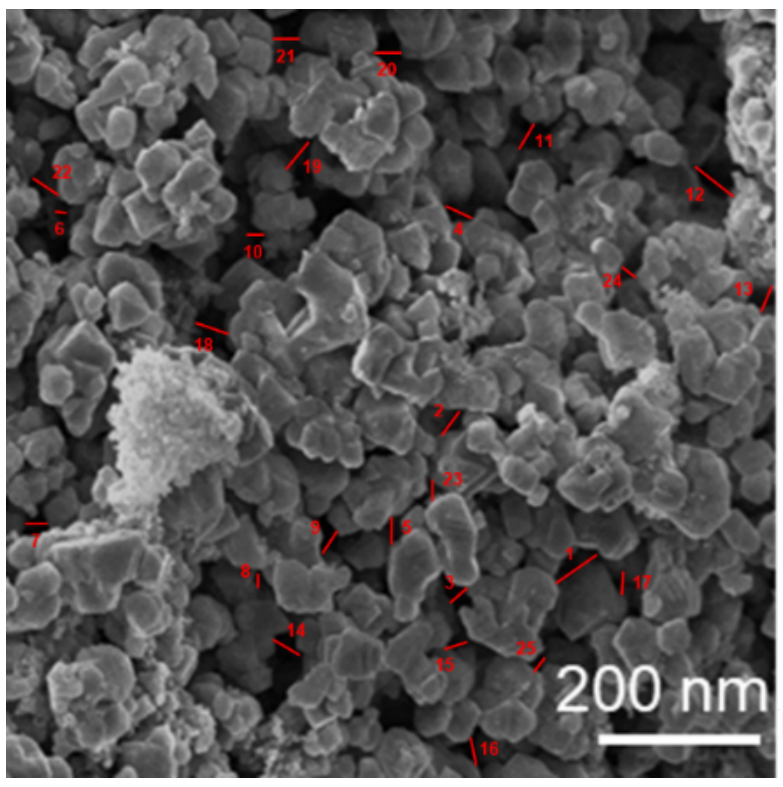

Figure S11. Representative cross-sectional SEM images of etched nanoporous thermosets illustrating the ImageJ-based method used to measure pore diameters. Red markers indicate individual pore diameters selected for statistical analysis. A minimum of 25 pores per sample (n ≥ 25) were measured across 2 independent images to ensure statistical representativeness. Scale bar: 200 nm.

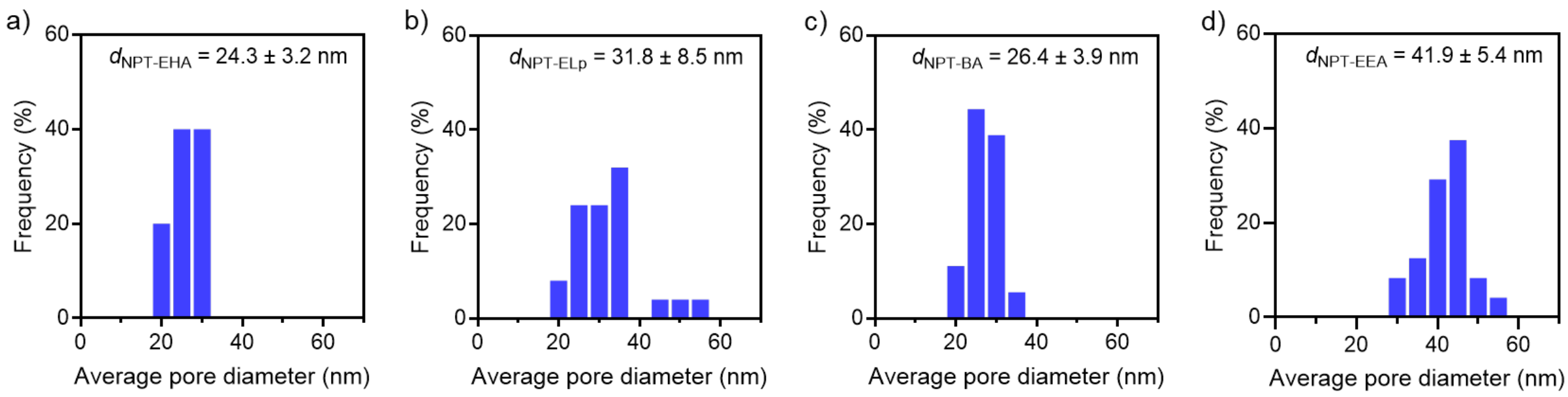

Figure S12. Pore diameter frequency distributions for etched nanoporous thermosets derived from statistical analysis of cross-sectional SEM images (ImageJ, n ≥ 25 pores per sample). Mean pore diameters of 24.3 nm, 31.8 nm, 26.4 nm, and 41.9 nm were determined for a) NPT-EHA, b) NPT-ELp, c) NPT-BA, and d) NPT-EEA, respectively. Dashed vertical lines indicate the mean value for each distribution. A minimum of 25 pores per sample (n ≥ 25) were measured across 2 independent images to ensure statistical representativeness.

Table S3. Average pore diameters of the nanoporous thermosets (NPTs) determined by statistical analysis of SEM images using ImageJ software.

Entry	Nanoporous monoliths (NPTs)	Average pore diameter (nm)
1	NPT-EHA	24.3 ± 3.2
2	NPT-ELp	31.8 ± 8.5
3	NPT-BA	26.4 ± 3.9
4	NPT-EEA	41.9 ± 5.4

Note: A minimum of 25 pores per sample ($n \geq 25$) were measured across 2 independent images to ensure statistical representativeness. Scale bar: 200 nm.

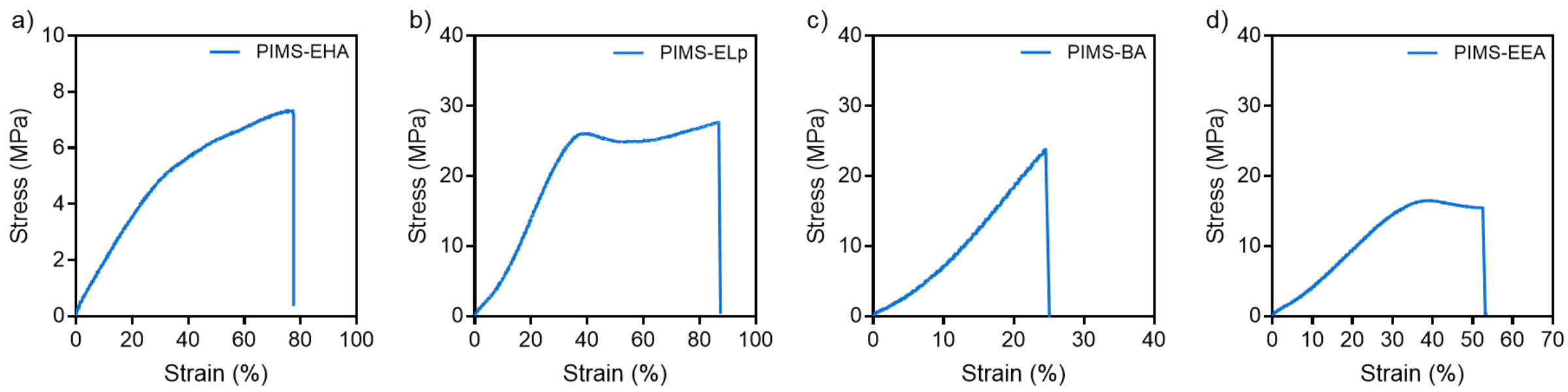

Figure S13. Representative stress–strain curves of the PIMS precursors prior to selective etching, demonstrating the mechanical robustness of the crosslinked networks before nanopore generation. a) PIMS-EHA, b) PIMS-ELp, c) PIMS-BA, and d) PIMS-EEA. Representative curves were selected from tensile measurements performed on n = 5 specimens per formulation.

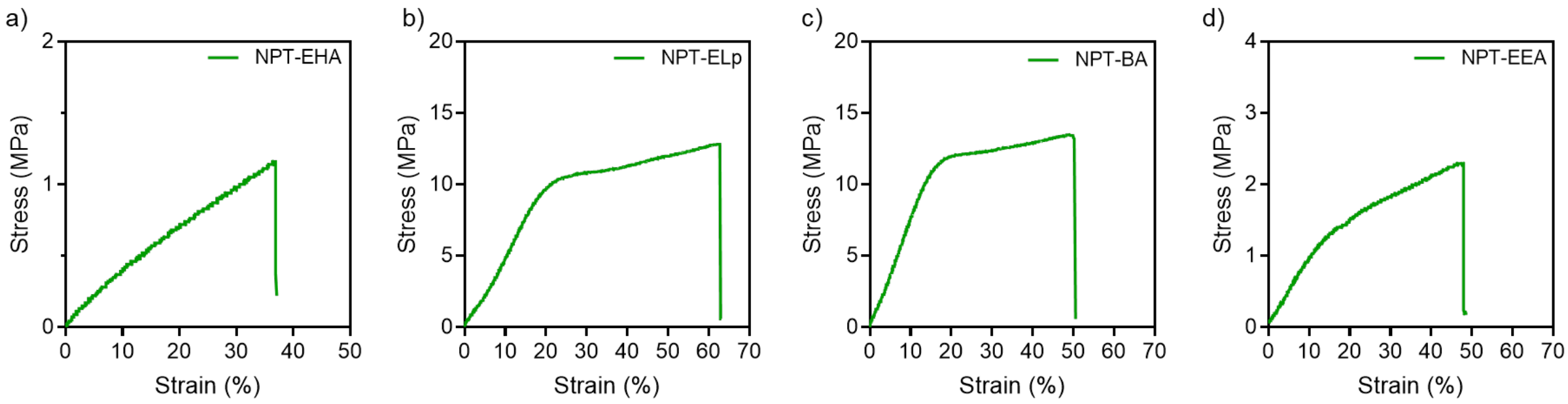

Figure S14. Representative stress–strain curves of the nanoporous thermosets after selective etching. The reduced stiffness reflects the introduction of nanoscale porosity following removal of the degradable macroCTA domains, while the materials retain overall mechanical integrity. a) NPT-EHA, b) NPT-ELp, c) NPT-BA, and d) NPT-EEA. Representative curves were selected from tensile measurements performed on n = 5 specimens per formulation.

Table S4. Mechanical properties of the as-printed PIMS mateirals before etching and the corresponding nanoporous thermosets after selective etching (NPTs), including Young's modulus, ultimate tensile strength, and strain at break. Values are reported as mean ± standard deviation from at least five independent measurements.

Type	Sample	Young's modulus (MPa)	Ultimate Tensile Strength (MPa)	Strain (%)
PIMS	EHA	39.7 ± 6.5	7.5 ± 0.4	79.7 ± 3.7
	ELp	91.9 ± 15.9	26.6 ± 0.7	86.9 ± 13.7
	BA	93.0 ± 20.8	27.4 ± 3.6	29.5 ± 5.9
	EEA	66.6 ± 7.4	14.4 ± 1.1	57.3 ± 9.0
NPT	EHA	5.9 ± 1.4	1.0 ± 0.1	36.8 ± 1.2
	ELp	50.0 ± 12.1	10.7 ± 1.8	61.6 ± 3.0
	BA	71.1 ± 8.7	13.5 ± 1.5	42.7 ± 11.3
	EEA	6.9 ± 0.3	1.9 ± 0.1	48.5 ± 1.6

Table S5. Optimization of LCD printing exposure parameters for the PIMS precursor resins. Different bottom-layer and normal-layer exposure times were evaluated to balance curing completeness and feature fidelity.

Entry	Bottom-layer exposure time (s)	Layer exposure time (s)	Printing quality
1	40	40	severe overcuring
2	40	30	slightly overcuring
3	40	20	good fidelity
4	40	10	incomplete curing
5	30	15	Undercured<br>poor adhesion on the building stage

Note: Resin compositions were held constant at 30 wt% macroCTA, 0.5 wt% TPO photoinitiator, and a vinyl monomer-to-crosslinker molar ratio of 4:1, with layer thickness and light intensity fixed at 100 μm and 3.5 mW cm^{-2}, respectively.

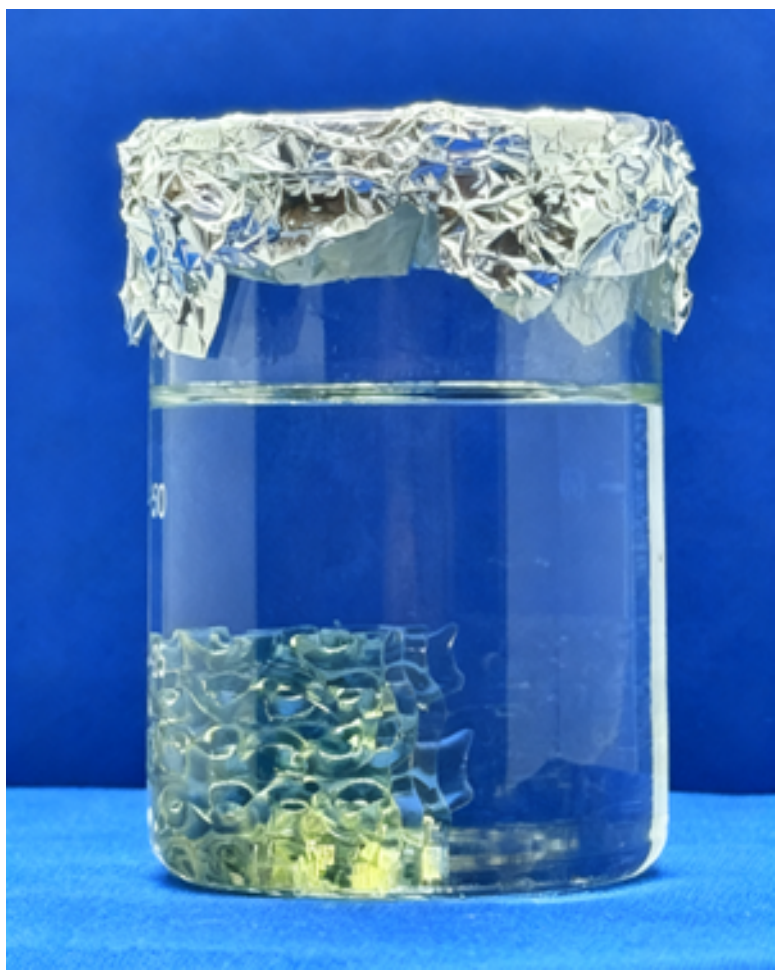

Figure S15. Selective etching procedure for 3D-printed complex objects. The 3D-printed gyroid structure of PIMS-EHA was soaked in 0.5M DBU/acetone solution to selectively remove P(LA-*co*-EHA)-macroCTA for 2 h.

Table S6. Gravimetric mass loss of complex 3D-printed architectures after selective etching via 0.5 M DBU in acetone.

Entry	Sample	Model	Theoretical mass loss (wt%)	Experimental mass loss (wt%)
1	PIMS-EHA	Gyroid	30	20.7± 2.2
2	PIMS-ELp	Schwarz P-cube	30	24.6 ± 2.4
3	PIMS-BA	UNSW letters	30	21.4 ± 1.7
4	PIMS-EEA	Vase	30	22.5 ± 1.5

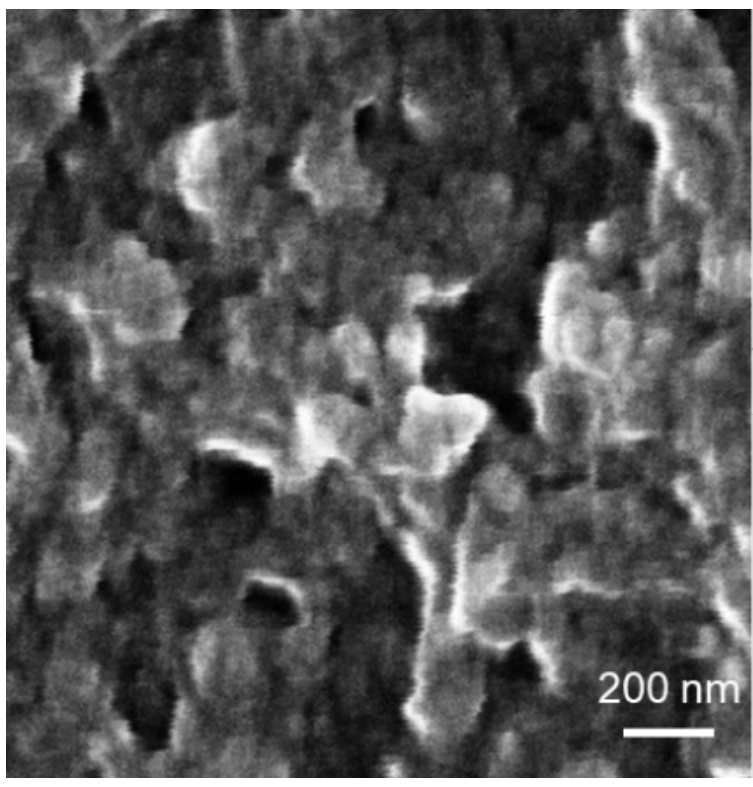

Figure S16. Cross-sectional SEM image of the etched PIMS-EHA monolith obtained by selective removal of P(LA-*co*-EHA)-macroCTA within 3D-printed gyroid PIMS precursor. Note: samples were coated with thin layer of Pt (~1 nm).

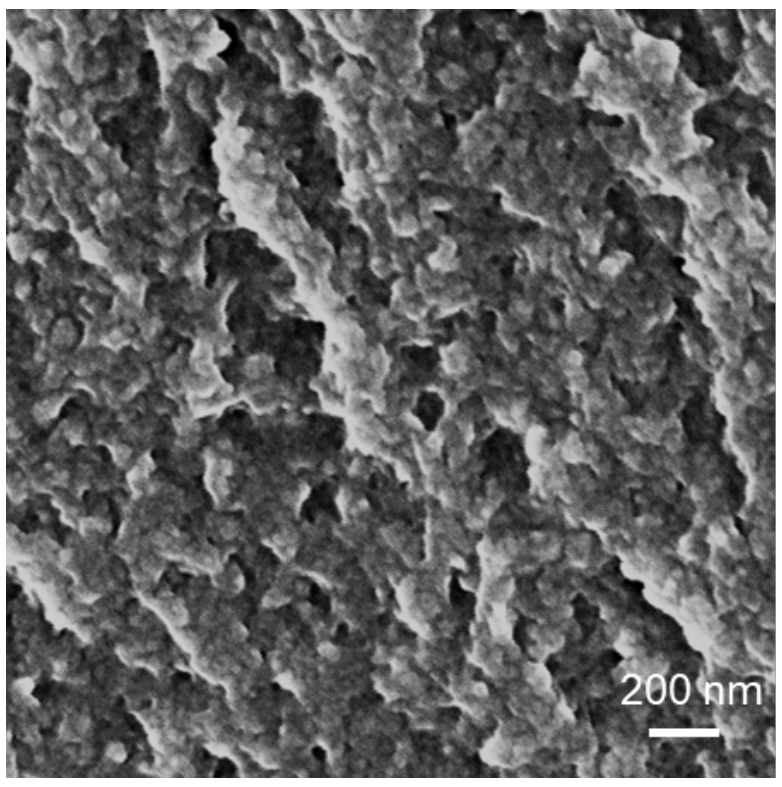

Figure S17. Cross-sectional SEM image of cross section of the etched PIMS-ELp monolith obtained by selective removal of P(ELp-*co*-BA)-macroCTA within 3D-printed Schwarz P-cube PIMS precursor. Note: samples were coated with thin layer of Pt (~1 nm).

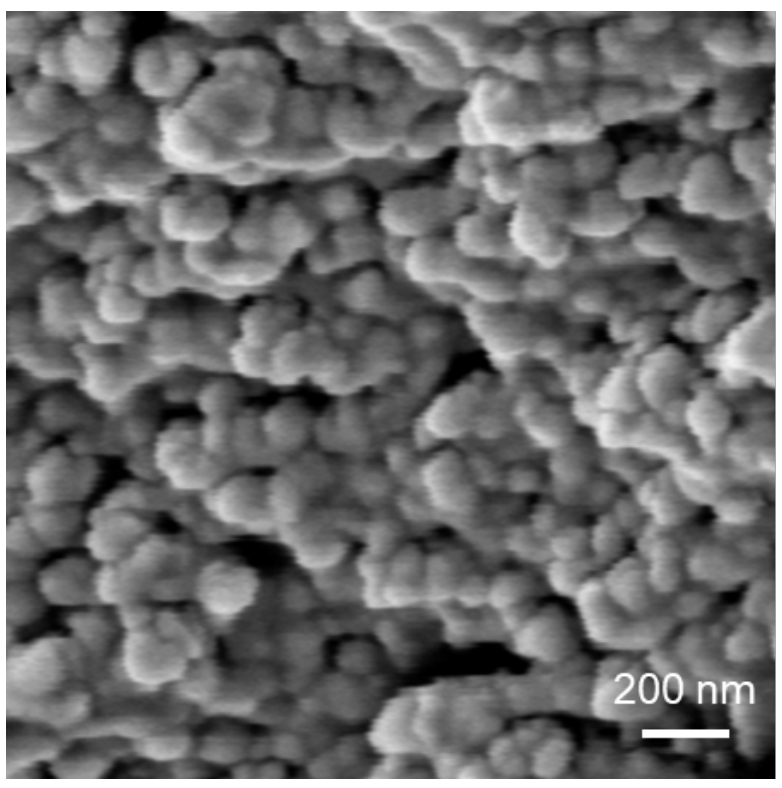

Figure S18. Cross-sectional SEM image of cross section of the etched PIMS-EEA obtained by selective removal of P(LA-*co*-EEA)-macroCTA within 3D-printed vase PIMS precursor. Note: samples were coated with thin layer of Pt (~1 nm).

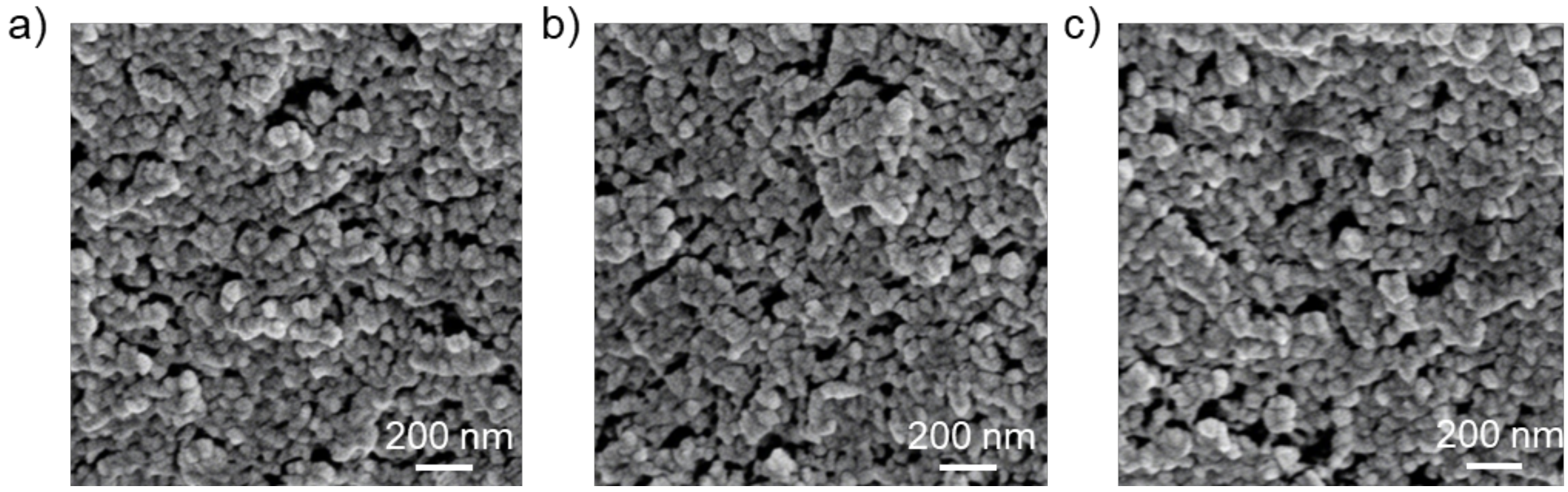

Figure S19. Cross-sectional SEM images collected from different regions of etched PIMS-EEA printed structures, showing comparable nanoporous morphologies throughout the printed cross-section without obvious pore discontinuities or dense interfacial regions. Note: samples were coated with thin layer of Pt (~1 nm).

Table S7. Quantitative dimensional analysis of 3D-printed architectures before and after selective etching, determined from optical images using ImageJ software.

Entry	3D-printed samples	Geometry	Shrinkage rate (%)
1	PIMS-EHA	Gyroid	10.2
2	PIMS-ELp	Schwarz P cube	7.2
3	PIMS-BA	UNSW letters	6.5
4	PIMS-EEA	Vase	6.8

Note: All measurements were performed in duplicate.
